# *Toxoplasma* Effector MAF1 Mediates Recruitment of Host Mitochondria and Impacts the Host Response

**DOI:** 10.1371/journal.pbio.1001845

**Published:** 2014-04-29

**Authors:** Lena Pernas, Yaw Adomako-Ankomah, Anjali J. Shastri, Sarah E. Ewald, Moritz Treeck, Jon P. Boyle, John C. Boothroyd

**Affiliations:** 1 Department of Microbiology & Immunology, Stanford University School of Medicine, Stanford, California, United States of America; 2 Department of Biological Sciences, University of Pittsburgh, Pittsburgh, Pennsylvania, United States of America; University of Georgia, United States of America

## Abstract

Recent information has revealed the functional diversity and importance of mitochondria in many cellular processes including orchestrating the innate immune response. Intriguingly, several infectious agents, such as *Toxoplasma*, *Legionella*, and *Chlamydia*, have been reported to grow within vacuoles surrounded by host mitochondria. Although many hypotheses have been proposed for the existence of host mitochondrial association (HMA), the causes and biological consequences of HMA have remained unanswered. Here we show that HMA is present in type I and III strains of *Toxoplasma* but missing in type II strains, both *in vitro* and *in vivo*. Analysis of F1 progeny from a type II×III cross revealed that HMA is a Mendelian trait that we could map. We use bioinformatics to select potential candidates and experimentally identify the polymorphic parasite protein involved, mitochondrial association factor 1 (MAF1). We show that introducing the type I (HMA^+^) *MAF1* allele into type II (HMA^−^) parasites results in conversion to HMA^+^ and deletion of *MAF1* in type I parasites results in a loss of HMA. We observe that the loss and gain of HMA are associated with alterations in the transcription of host cell immune genes and the *in vivo* cytokine response during murine infection. Lastly, we use exogenous expression of MAF1 to show that it binds host mitochondria and thus MAF1 is the parasite protein directly responsible for HMA. Our findings suggest that association with host mitochondria may represent a novel means by which *Toxoplasma* tachyzoites manipulate the host. The existence of naturally occurring HMA^+^ and HMA^−^ strains of *Toxoplasma*, *Legionella*, and *Chlamydia* indicates the existence of evolutionary niches where HMA is either advantageous or disadvantageous, likely reflecting tradeoffs in metabolism, immune regulation, and other functions of mitochondria.

## Abstract 2

The intracellular human protozoan parasite *Toxoplasma gondii* uses a novel secreted protein to recruit host mitochondria and alter the host's response to infection.

## Author Summary

Recent discoveries have revealed the remarkable functional diversity of mitochondria in roles other than energy production, including an integral role for mitochondria and their dynamics in the regulation of the innate immune response. Interestingly, host mitochondria are recruited to the membranes that surround certain intracellular bacteria and parasites during infection. To date, how and why this phenomenon occurs has been a mystery, although it has been proposed to provide a metabolic benefit to the microbes. Here we identify mitochondrial association factor 1 (MAF1) as the parasite protein that mediates the association between the protozoan pathogen *Toxoplasma* and host mitochondria during infection. We show that MAF1 is needed to recruit host mitochondria to the *Toxoplasma-*containing vacuole and that this process is associated with changes in the immune response in infected cells and animals. These findings show that recruitment and association with host mitochondria is an important means by which intracellular pathogens interface with their host. We also find that the cost–benefit outcome of altering mitochondrial function might differ between strains depending on the precise niche in which they evolved; for infectious agents, these differences likely reflect different host organisms.

## Introduction

Mitochondria are highly dynamic organelles that, besides producing energy and regulating apoptotic and Ca^2+^ signals, play a key role in orchestrating several aspects of cell function and behavior including autophagy, apoptosis, and immune signaling [1]–[3]. Interestingly, recent evidence indicates that mitochondrial dynamics, including distribution, size, and shape, are linked to altered regulation of certain innate immune responses [4],[5].

Given this diversity of function, it is no surprise that mitochondria have recently emerged as central to host–pathogen interactions, with an increasing number of viruses and bacteria having been shown to manipulate host mitochondria in their roles in apoptosis, energy production, and immune function. For example, the hepatitis C virus protease NS3/4a cleaves the N-terminal fragment of the mitochondrial antiviral signaling protein (MAVS), rendering infected cells unable to induce interferon (IFN) production [6], and *Helicobacter pylori* vacuolating cytotoxin (VacA) engages the machinery involved in regulation of mitochondrial fission to induce apoptosis [7].

In light of these newly established roles for mitochondria and their targeting by different microbial effector proteins, it is interesting that during infection with certain pathogens host mitochondria associate with and appear sequestered at the vacuole in which the microbes reside. This phenomenon has been previously reported during infection with the bacteria *Legionella pneumophila* [8] and *Chlamydia psittaci* [9] and the parasite *Toxoplasma gondii* [10], and this occurs both in mammalian (*T. gondii*, *L. pneumophila*, and *C. psittaci*) and protozoan host cells (*L. pneumophila* in *Hartmannella vermiforis* [11]). Although the mechanism and functional consequence of this association during infection with these bacteria remain unknown, it has been reported that this association is species-specific [9]—for example, *C. psittaci*, but not *Chlamydia trachomatis*, intimately associates with mitochondria. In the case of *T. gondii*, three predominant, clonal lineages have been described based on genotyping performed on isolates from human and animal infections in North America and Europe [12]. Referred to as type I, type II, and type III, these lineages differ widely in a number of phenotypes including migratory capacity [13], cytokine production in infected cells [14],[15], and virulence in mice [16]. Whether these lineages also differ in their ability to recruit host mitochondria, however, has not previously been assessed.

Since the initial discovery of host mitochondrial association (HMA) in macrophages infected with (type I) *Toxoplasma* tachyzoites in 1972 [10], the question of what parasite factor mediates this association has been of intense interest. Previous work suggested that *Toxoplasma* HMA was mediated by *Toxoplasma* rhoptry protein 2 (ROP2) [17]. However, subsequent work showed that parasites lacking ROP2 expression were indistinguishable from wild-type parasites in their ability to recruit host mitochondria, reopening the question of what factors mediated the association [18]. The functional significance of the recruitment of mitochondria to the *Toxoplasma* vacuole also remained a conundrum. Although HMA has long been assumed to reflect a crucial means by which the parasite acquires key metabolites, only limited evidence has emerged to support this hypothesis [19],[20]. Given our expanded understanding of the diverse roles performed by mitochondria, however, we hypothesized that HMA confers a selective advantage to the various infectious agents where it has been described and that mitochondrial functions other than metabolism might also contribute to this advantage.

Here, we report that HMA is strain-specific in *Toxoplasma* and that it is mediated by a novel, secreted parasite factor, mitochondrial association factor 1 (MAF1), which differs in sequence, gene copy number, and expression between the three canonical strains. We demonstrate that MAF1 is necessary and sufficient for HMA and go on to show that during *Toxoplasma* infection, HMA is associated, *in vitro* and *in vivo*, with substantially altered levels of cytokines. These results demonstrate that HMA is an additional means by which *Toxoplasma* tachyzoites interface with the immune signaling of the host and suggest that, in addition to possible metabolic roles, HMA may present a novel strategy for subversion of host immune signaling by a pathogen.

## Results

### *Toxoplasma* Association with Host Mitochondria Is Strain-Specific

**Figure 1:**
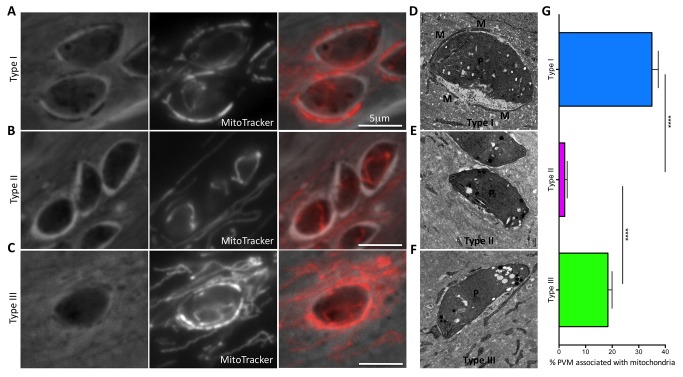
HMA in *Toxoplasma*-infected cells is strain-specific. (A–C) HFFs were labeled for 30 min at 37°C with 50 nM MitoTracker and then infected with (A) type I (RH), (B) type II (ME49), or (C) type III (CEP) strains of *T. gondii*. Cells were fixed 4 hpi and visualized by fluorescence microscopy. Phase, fluorescence (MitoTracker), and merged images are shown for each type. Scale bar, 5 µm. (D–F) Transmission electron micrographs depicting the PVM surrounding (D) type I, (E) type II, and (F) type III parasites grown in HFFs. Cells were fixed and processed for electron microscopy 4 hpi. Host mitochondria are indicated by M and parasites by P. (G) Percentage of the PVM associated with mitochondria in type I, II, and III vacuoles as determined by ImageJ analysis of electron micrographs (*n* = 20 for each). Values shown are mean ± SEM. ****p<0.0001 using an unpaired *t* test.

Given that the three predominant strains of *Toxoplasma* differ widely in a number of immune-related phenotypes [21] and that mitochondria have recently been shown to play a key role in orchestrating the cellular immune response to viral infection [22],[23], we hypothesized that HMA might differ between these canonical strains. To investigate this possibility, human foreskin fibroblasts (HFFs) were labeled with MitoTracker and infected with representative type I (RH), type II (Me49), or type III (CEP) strains of *T. gondii*. At 4 h postinfection (hpi), the type I and III tachyzoites showed the canonical, intimate, and extensive association of host mitochondria at the parasitophorous vacuole membrane (PVM)–host interface, whereas type II parasites showed little if any HMA (Figure 1A–C). To quantify and expand on these findings, the percentage of the PVM associated with host cell mitochondria was measured in electron micrographs of HFFs 4 hpi with type I, II, and III parasites (Figure 1D–F). The results showed that ∼35% and ∼18% of the PVM is tightly associated with host cell mitochondria in type I and III strains, respectively, in contrast to <2% of the PVM in infections with type II parasites (Figure 1G). Although host endoplasmic reticulum (ER) has also been reported to associate with the PVM [24], the abundance and size of the ER meant we were unable to confidently assess or quantify such association and so cannot comment on which strains of *Toxoplasma* do or do not exhibit this trait.

To ensure the generality of the HMA^−^ phenotype in type II strains, HMA was also assessed in an independent type II strain, Pru, which was isolated in France (Me49 was isolated in the United States of America); Pru was also found to be HMA^−^ (Figure S1), demonstrating that the HMA^−^ phenotype is not an isolate-specific property of Me49. Similarly, to determine whether HMA differed among cell lines of different mammalian and tissue origins, we tested the phenotype in murine macrophages and Madin–Darby canine kidney cells and obtained the same results as in the HFFs: type II was consistently HMA^−^, whereas types I and III were HMA^+^ (unpublished data). To determine if HMA occurs during *in vivo* infection, peritoneal exudate cells (PECs) were isolated from mice infected with type I and type II parasites and processed for electron microscopy analysis. Quantification of HMA in infected PECs (Figure S2) confirmed that HMA occurs in animals infected with type I but not type II strains. These results show that recruitment of host mitochondria to the *Toxoplasma* PVM is a strain-specific property of Toxoplasma *in vitro* as well as *in vivo*.

### HMA Phenotype Maps to *Toxoplasma* Chromosome II

Previous work has utilized genetic crosses between the canonical *Toxoplasma* lineages to map the loci responsible for major phenotypic differences [25]. We sought to test our hypothesis that strain-specific HMA had a genetic basis by utilizing F_1_ progeny from a type II×III cross [26], as these lineages differ in their ability to recruit mitochondria and their progeny have been previously genotyped at 135 different markers [27].

**Figure 2:**
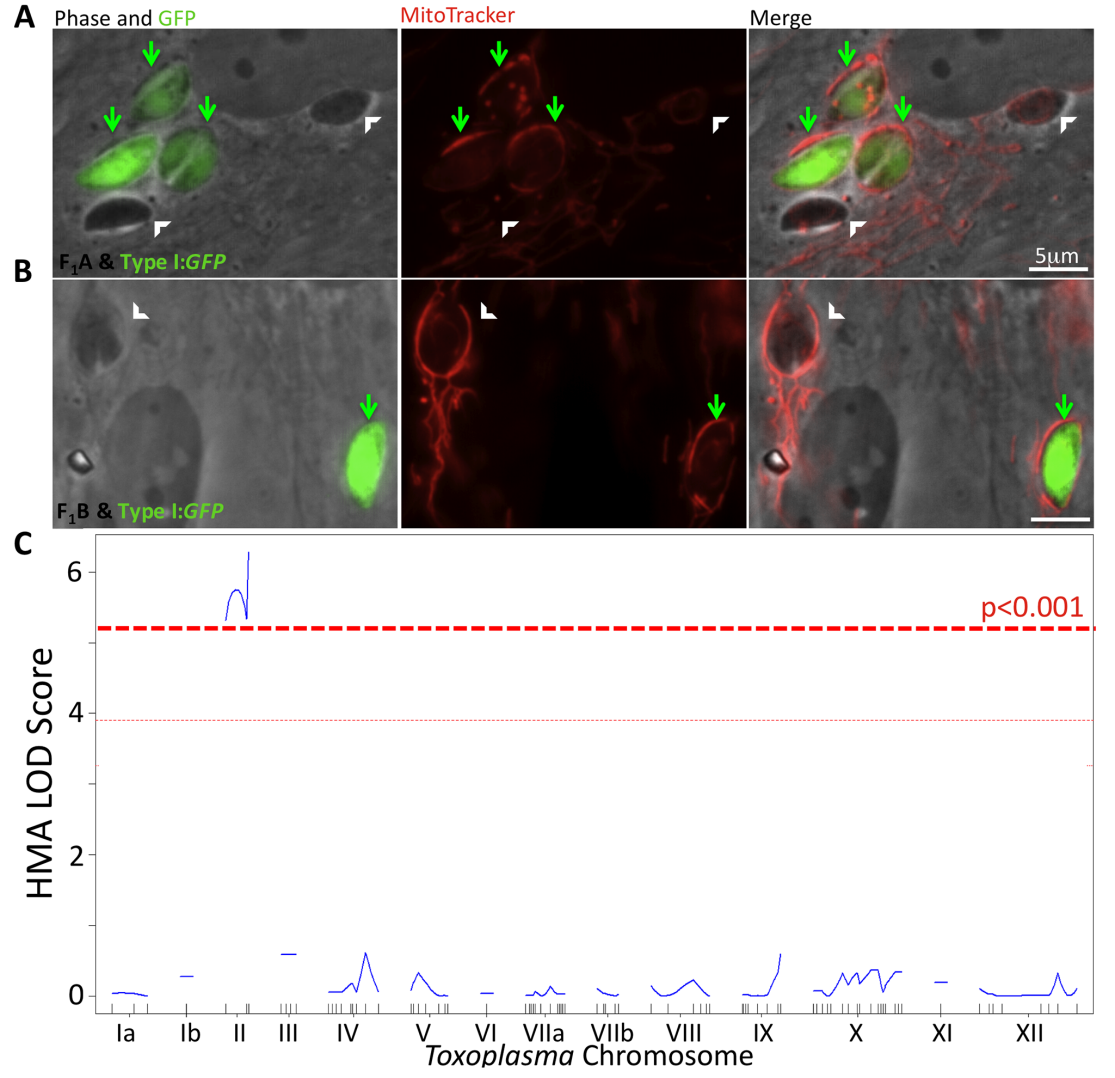
HMA phenotype maps to *Toxoplasma* chromosome II. HFFs were labeled with MitoTracker and then co-infected with a type I GFP^+^ strain (arrows) and either of two nonfluorescent F_1_ progeny (white arrowheads), (A) STG2 (F_1_A, top panel) and (B) E7 (F_1_B, bottom panel). Cells were fixed 4 hpi and visualized by fluorescence microscopy. Phase and green fluorescence (GFP) are shown in the first image of each panel. Next to that is the red fluorescence (MitoTracker) followed by a merged image of the two. Scale bar, 5 µm. (C) LOD Score plot indicates the log-likelihood of association of HMA with markers across the entire *Toxoplasma* genome in numerical chromosome order.

To facilitate HMA screening in the F1 progeny, we first determined whether co-infection with an HMA^−^ strain and HMA^+^ strain could alter the HMA phenotype of either parasite. The results showed that co-infection of HFFs with strains that differ in HMA did not affect the phenotype: type II parasites were within HMA^−^ vacuoles, whereas types I and III showed efficient HMA (Figure S3). To reliably determine the HMA phenotype of the F1 progeny, therefore, HFFs were co-infected with each of the 34 F_1_ progeny and a GFP expressing HMA^+^ control. This allowed for a direct comparison and qualitative assessment of the HMA phenotype between each progeny and the HMA^+^ control within the same cell. In all, 15 progeny were HMA^−^, 18 were HMA^+^, and one was indeterminate; example HMA^+^ and HMA^−^ progeny are shown in Figure 2A and Figure 2B, respectively. To identify the genomic region(s) mediating HMA, we searched for association between the previously mapped *Toxoplasma* genetic markers [27] and the HMA phenotype and found a highly significant co-segregation between HMA and chromosome II (LOD ∼6.3; *p*<0.001, Figure 2C); only F_1_ progeny having type III alleles at chromosome II were HMA^+^ (Figure S4). To refine the preliminary mapping, we analyzed the genotypes of two progeny with recombinations on chromosome II, E7 and STG2 (Figure S4, boxed region), which indicated that the region containing the parasite factor that mediates HMA is in the interval between the left end of chromosome II and marker KT-L379A, a maximum size of ∼1.2 Mb.

### TGGT1_053770 Is a Novel Secreted *Toxoplasma* Protein and Candidate Mediator of HMA

**Figure 3:**
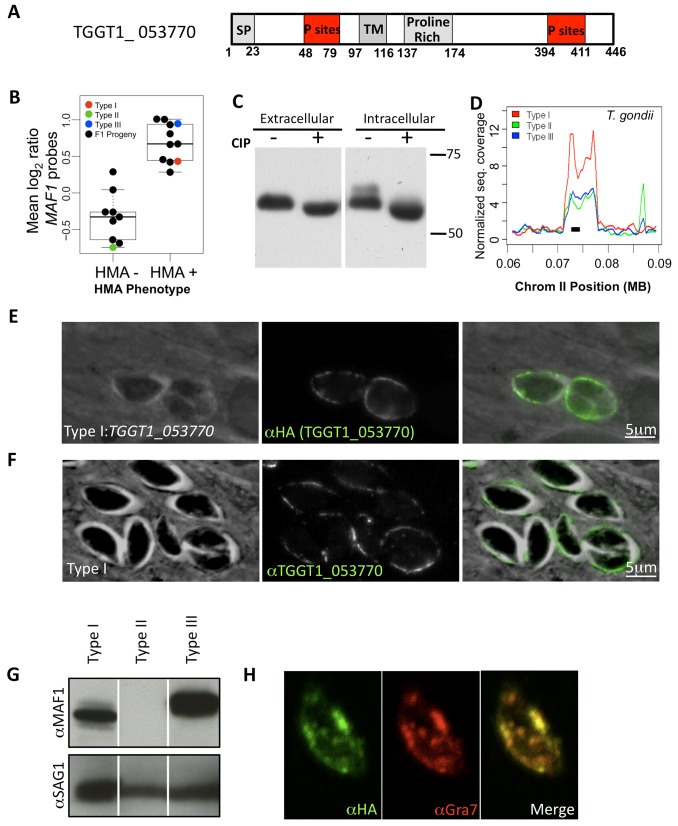
TGGT1_053770 is a novel secreted *Toxoplasma* protein and candidate mediator of HMA. (A) Schematic diagram of the TGGT1_053770 protein. Signal peptide (SP), TM domain, and phosphorylation sites (P sites) are indicated where predicted by SignalP 4.0 (http://www.cbs.dtu.dk/services/SignalP/), TMHMM v. 2.0 (http://www.cbs.dtu.dk/services/TMHMM/), and ToxoDB (www.toxodb.org). (B) Microarray expression values (log2 ratios of sample intensity over control intensity) for 17 of the HMA-phenotyped progeny (black data points). Red, green, and blue data points represent expression values for type I (RH; HMA+), II (PDS; HMA^−^), and III (CTG; HMA+) strains, respectively. Data shown are averages across 10 probes on the cDNA expression array that map to the *MAF1* locus. (C) Lysates from extracellular type I parasites and type I–infected HFFs (intracellular) were treated with and without CIP and loaded in each lane. The membrane was probed with anti-HA antibody conjugated to peroxidase. (D) Normalized sequence coverage (*y*-axis) reflective of the number of copies of TGGT1_*053770* on chromosome II (*x*-axis) for types I (GT1), II (ME49), and III (VEG). The region putatively encoding TGGT1_053770 is indicated by a black bar. (E) Type I parasites expressing HA-tagged TGGT1_053770 were added to HFFs, and cultures were fixed 6 hpi. Following permeabilization, TGGT1_053770 was visualized using anti-HA antibodies. Scale bar, 5 µm. (F) Type I parasites were added to HFFs, and cultures were fixed 6 hpi. Following permeabilization, TGGT1_053770 was visualized using polyclonal anti-TGGT1_053770 mouse sera. Scale bar, 5 µm. (G) Western blot showing expression of TGGT1_053770 in type I, II, III and type II parasites. Blots were probed with antibodies to recombinant TGGT1_053770 (upper panels), then probed for surface antigen SAG1 as a loading control (lower panels); white vertical lines indicate that the order of the lanes shown is different from the original loading of the gel. Note that the product of the type III allele has a reproducibly slower electrophoretic mobility than type I. (H) Syringe-lysed parasites were plated on coverslips and fixed, labeled with anti-HA (TGGT1_053770) and anti-GRA7 antibodies, and visualized using confocal microscopy.

To choose among the list of 153 genes present in the region identified on chromosome II as possibly involved in HMA, we used a candidate gene approach based on properties predicted for the parasite factor. HMA occurs at the PVM and is strain-specific; thus, we used bioinformatics to first identify genes that encoded a predicted signal peptide and a transmembrane (TM) domain, and had different expression levels between HMA^+^ (types I and III) and HMA^−^ (type II) strains. Of the 10 genes that fulfilled these criteria, the type I gene *TGGT1_053770* (denoted in ToxoDB v7.2; as *TGME49_020950* in type II and *TGVEG_051550* in type III; Figure 3A) stood out as the most promising for multiple reasons: (1) This gene had been previously identified in proteomic studies as a secreted *Toxoplasma* antigen [28], suggesting that it was capable of interacting with the host cell; (2) microarray data for 17 of the progeny phenotyped for HMA showed that this transcript was of generally higher abundance in the 9 F_1_ progeny that were HMA^+^ relative to the 8 F_1_ progeny that were HMA^−^ (Figure 3B) [29]; (3) we had also observed in prior studies that the protein encoded by *TGGT1_053770* is heavily phosphorylated in infected cells relative to extracellular parasites [30], a further indication that it might be secreted into the PV or host cell, and we confirmed postinvasion phosphorylation of TGGT1_053770 by comparing electrophoretic migration after addition of phosphatase in intracellular and extracellular parasite lysates (Figure 3C); and (4) lastly, as with *Toxoplasma ROP5*, which is crucial for type I virulence in mice [31], there is precedent for gene family expansions playing a key role in interfacing with host immune defenses [32],[33] and determining pathogen host range [34]. The *TGGT1_053770* locus is expanded in *T. gondii*: based on raw genomic sequence coverage, type I (GT1) has ∼6–10 copies, whereas types II (ME49) and III (VEG) have ∼4–5 copies (Figure 3D).

To determine if the protein encoded by *TGGT1_053770* traffics to the PVM, a property expected of a protein mediating HMA, HFFs were infected with type I parasites engineered to ectopically express an N-terminally HA-tagged version of the protein driven by its own promoter and with the HA-tag placed just downstream of the putative signal peptide. Using anti-HA antibody, the tagged protein was indeed found to localize at the PVM (Figure 3E). This localization was confirmed by raising antibodies to recombinant TGGT1_053770 and showing that these antibodies likewise stained the PVM of cells infected with type I parasites (Figure 3F). There was near-perfect co-localization of the anti-HA antibodies and anti-TGGT1_053770 antibodies in cells infected with type I parasites expressing *TGGT1_053770*, showing that the N-terminal tagging did not detectably alter the protein's trafficking in the infected cells (unpublished data). Given that the PVM and intracellular staining pattern was consistent with that of a dense granular protein, we also looked with antibodies to dense granule protein 7 (GRA7) and anti-HA antibodies and found that there was co-localization between these two proteins in extracellular parasites expressing the *TGGT1_053770* transgene (Figure 3H); hence, *TGGT_053770* appears to encode a dense granule protein.

To compare expression of this locus between the archetypal strains, Western blot analysis of extracellular lysates of type I, II, and III tachyzoites was performed using anti-TGGT1_053770 antibodies. The results showed that although expression levels were comparable in HMA^+^ type I and III parasites (albeit the type III protein migrates slightly but reproducibly more slowly), type II parasites showed no detectable expression (Figure 3G), consistent with the microarray data in Figure 3D. To determine if this failure to detect a signal in lysates from type II parasites might be due to a gross difference between the allele in this strain relative to TGGT1_053770, we PCR-amplified and sequenced the locus from RH (type I) and Me49 (type II) genomic DNA. Although the tandem duplications of this locus make it difficult to determine the true extent of sequence variation within and between strains, the results showed a 94.5% predicted amino acid identity between the two types (Figure S5), making it very unlikely that polyclonal antisera raised to one would not react with the other.

Collectively, the data above indicate that *TGGT1_053770* has the expected properties of a parasite mediator of HMA in being a tandemly repeated locus that differs in sequence, copy number, and expression between strains and that encodes a novel dense granule phosphoprotein found at the PVM.

### TGGT1_053770 (MAF1) Mediates *Toxoplasma* HMA

**Figure 4:**
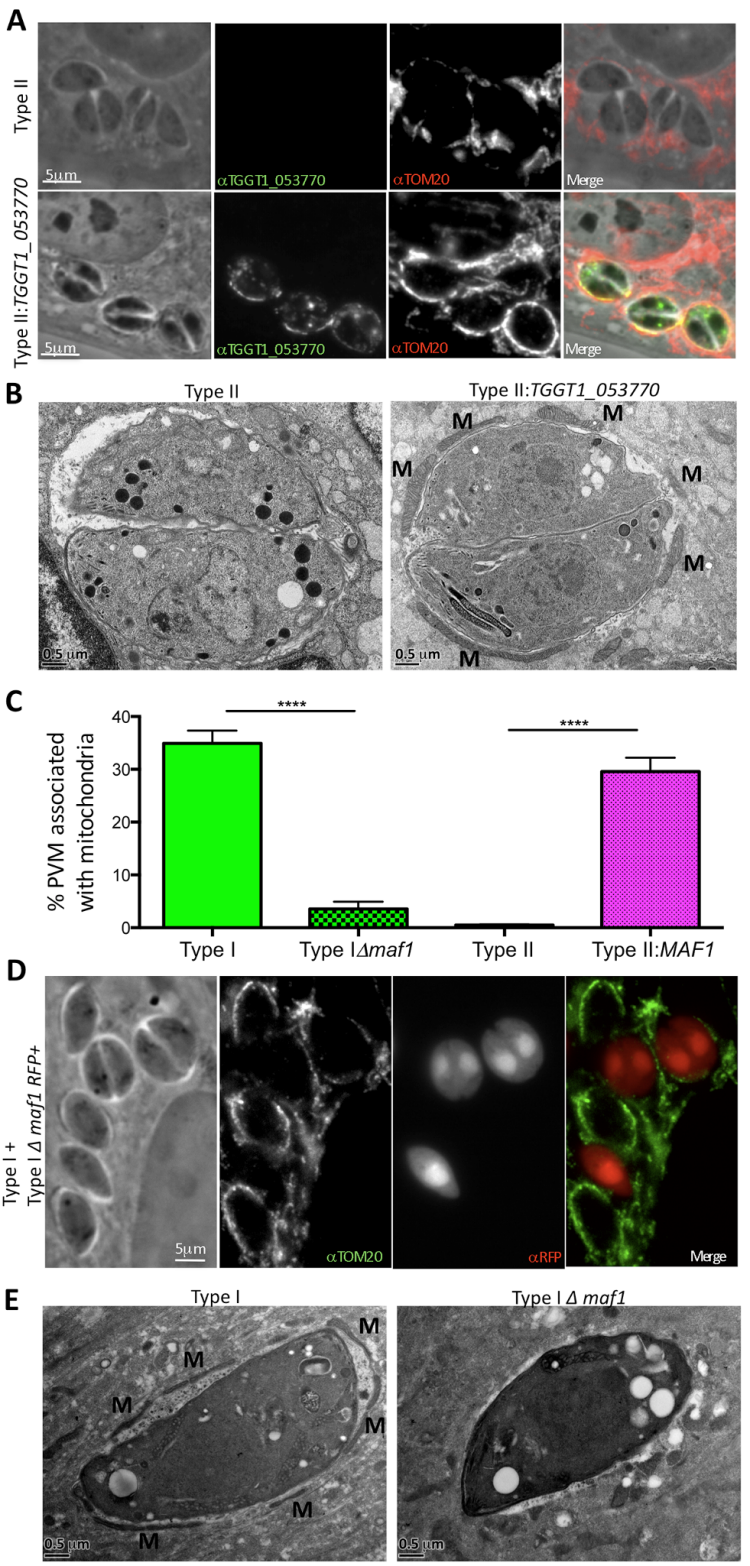
MAF1 (TGGT1_053770) mediates *Toxoplasma* HMA. (A) Type II or type II parasites expressing an N-terminally HA-tagged *TGGT1_053770* transgene were used to infect HFFs. Cells were fixed 12 hpi, and following permeabilization, TGGT1_053770 was visualized by immunofluorescence microscopy using polyclonal mouse sera raised against recombinant TGGT1_053770; host mitochondria were visualized using rabbit anti-TOM20 antibodies. (B) Transmission electron micrographs depicting the PVM surrounding type II and type II:*TGGT1_053770* parasites grown in mBMDMs. Cells were fixed and processed for electron microscopy 6 hpi. Host mitochondria are indicated by M. (C) Percentage of the PVM associated with mitochondria in HFFs 4 hpi with type I and type I:*Δmaf1*, and BMMs 17 hpi with type II and type II:*MAF1* vacuoles as determined by ImageJ analysis of electron micrographs (*n* = 20 for each). Values shown are mean ± SEM. ****p<0.0001 using an unpaired *t* test. (D) Type I and type I:*Δmaf1* parasites (expressing RFP) were used to infect HFFs, which were then fixed 4 hpi. Following permeabilization, host mitochondria were visualized using rabbit anti-TOM20 antibodies. (E) Transmission electron micrographs depicting the PVM surrounding type I and type I:*Δmaf1* parasites grown in HFFs. Cells were fixed and processed for electron microscopy 4 hpi. Host mitochondria indicated by M. Scale bars are 5 µm (A and C) and 0.5 µm (B and D).

To directly test the hypothesis that TGGT1_053770 mediates HMA, we infected HFFs with type II parasites or type II parasites engineered to express the N-terminally HA-tagged type I allele of this gene (referred to here as type II:*MAF1*). At 12 hpi, cells were fixed and stained with anti-TGGT1_053770 polyclonal sera along with antibodies to Translocase of the Outer Membrane 20 (TOM20), a marker of host mitochondria. The results (Figure 4A) showed that expression of TGGT1_053770 in type II tachyzoites is associated with a dramatic gain in HMA, with the recruited mitochondria being specifically associated with the portions of the PVM that stained with anti-TGGT1_053770. Electron microscopy (Figure 4B) confirmed that the HMA^+^ phenotype of type II:*TGGT1_053770* parasites results in a marked increase in the levels of mitochondrial recruitment; on average, ∼30% of the PVM surface appears associated with host mitochondria when infecting with these parasites compared to <2% with type II parasites (Figure 4C). The results above show that *TGGT1_053770* encodes a novel protein that is sufficient for recruitment of host mitochondria to the vacuole containing type II *Toxoplasma* parasites and confirm the role of TGGT1_053770 in mediating HMA. We will hereafter refer to TGGT1_053770 as MAF1.

To further explore the role of MAF1 in HMA, type I parasites lacking *MAF1* were generated (*Δmaf1*) and verified as deleted for *MAF1* via PCR, Western blot analysis, and microscopy (Figure S6). Co-infection experiments using type I and type I:*Δmaf1* parasites showed the predicted HMA dependency on MAF1 (Figure 4D); type I parasites exhibit HMA in contrast to the clear loss of HMA in type I:*Δmaf1* parasites. Electron microscopy (Figure 4E) was used to quantify the difference in HMA: only ∼3% of the PVMs surrounding type I:*Δmaf1* were associated with host mitochondria, in contrast to ∼35% for type I PVMs (Figure 4C). These results indicate that MAF1 is necessary for HMA during type I infection.

**Figure 5:**
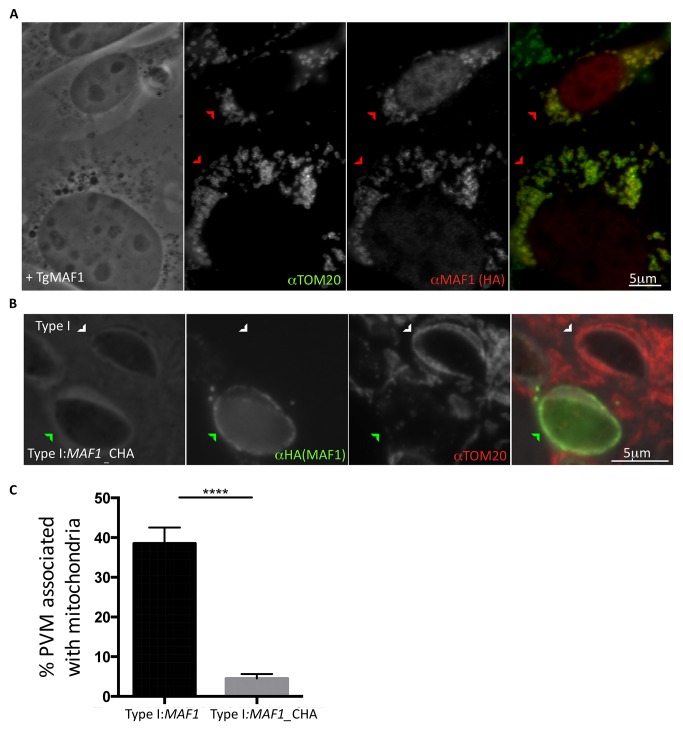
Exposure of the MAF1 C-terminus is essential for its role in HMA. (A) MEFs retrovirally transduced for expression of MAF1 were fixed, and following permeabilization, MAF1 was visualized by immunofluorescence microscopy using HA antibody, and host mitochondria were visualized using rabbit anti-TOM20 antibodies. Red arrowheads indicate MEFs expressing the transgene. Scale bar, 5 µm. (B) HFFs were co-infected with type I (white arrowhead) and type I parasites expressing C-terminally HA-tagged MAF1 (MAF1_CHA; green arrowhead) and fixed 4 hpi. Following permeabilization, MAF1 was visualized using anti-HA antibodies and mitochondria using anti-TOM20 antibody. Scale bar, 5 µm. (C) Percentage of PVM associated with mitochondria in HFFs 4 hpi with type I:MAF1 and type I:MAF1_CHA as determined by ImageJ analysis of electron micrographs (*n* = 23 for each). Values shown are mean ± SEM. ****p<0.0001.

We next sought to investigate whether MAF1 is directly responsible for HMA or whether a parasite partner protein is involved. To do this, we imaged mouse embryonic fibroblasts (MEFs) retrovirally transfected with an expression construct encoding N-terminally HA-tagged MAF1. IFA analysis of a transfected population showed a clear co-localization of MAF1 with TOM20, a mitochondrial membrane marker (Figure 5A), indicating that MAF1 is directly responsible for HMA.

### Exposure of the C-Terminus of MAF1 Is Essential for Its Role in HMA

Although BLAST searches yielded no clues as to the coding function or region of MAF1 responsible for HMA, we hypothesized that during *Toxoplasma* infection the predicted TM domain anchors MAF1 at the PVM, allowing for the MAF1 C-terminal domain to be exposed to the host cell cytosol, where it tethers host mitochondria. To test this hypothesis, type II parasites were engineered to express MAF1 bearing a C-terminal HA epitope tag (MAF1_CHA). The HA tag served a dual purpose: to allow for recognition of the expressed transgene as well as function as a steric block if an exposed C-terminus is necessary for HMA. The results showed that the type II:*MAF1_CHA* parasites remain HMA^−^, suggesting a native C-terminus is necessary for MAF1 function (unpublished data). Surprisingly, in type I parasites engineered to express *MAF1_CHA*, the C-terminally HA-tagged MAF1 acts as a dominant negative; its expression results in a dramatic loss of HMA at 4 hpi even though it correctly localizes to the PVM (Figure 5B and C). These data suggest that the C-terminal domain of MAF1 is key to its role in HMA, although we cannot exclude an indirect effect of the C-terminal epitope tag.

### MAF1 Induces Changes in Mitochondrial Morphology and Is Associated with an Altered Host Cell Response to *Toxoplasma*

The identification of MAF1 as the molecule that *Toxoplasma* uses to mediate HMA enabled us to next address the functional consequences of HMA in the host–pathogen interaction. HMA has been hypothesized to serve as a source of metabolites for the parasite, and previous work has shown that *Toxoplasma* scavenges host-derived mitochondrial lipoic acid [19]. To determine the role of HMA during tachyzoite growth within a host cell, type I versus type I:*Δmaf1* proliferation was compared in a competitive growth assay in HFFs *in vitro*. At 7–10 d postinfection (dpi), we observed no significant growth disadvantage in type I:*Δmaf1* parasites due to the loss of HMA. A similar comparison of growth of type II versus type II:*MAF1* parasites also yielded no reproducible change in their relative numbers 7–10 dpi (unpublished data). Collectively, these data support the conclusion that HMA does not result in growth differences between type I and type II parasites in a nutritionally replete environment, although this does not exclude a metabolic role for HMA.

**Figure 6:**
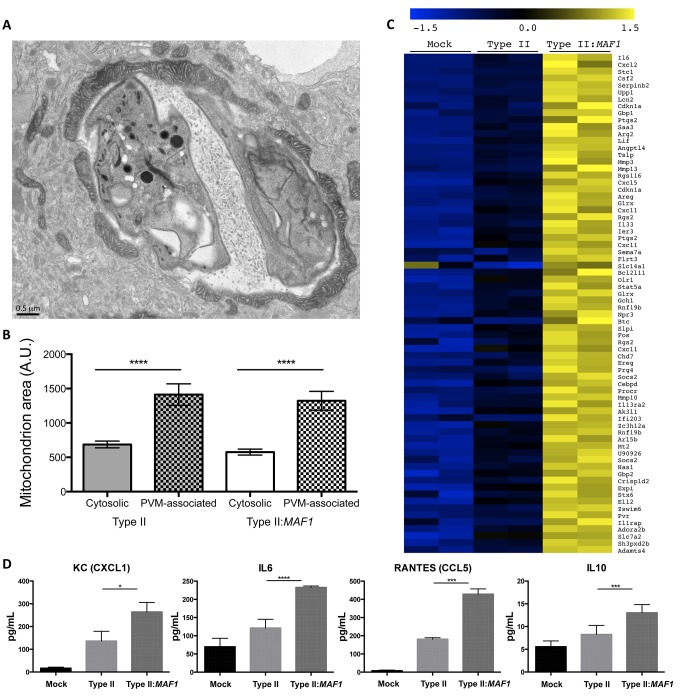
MAF1 induces changes in mitochondrial morphology and is associated with an altered host cell response to *Toxoplasma*. (A) Transmission electron micrograph of a mBMDM infected 17 hpi with type II:*MAF1* parasites. Cells were fixed and processed for electron microscopy. Scale bar, 0.5 µm. (B) Cross-sectional area of cytosolic and PVM-associated mitochondria during type I and type II:*MAF1* infection as determined by EM analysis (*n* = 20 for each). Values are mean ± SEM. ****p<0.0001. (C) MEFs were mock-infected or infected with type II or type II:*MAF1 Toxoplasma s*trains at an MOI of 5. Then, 8 hpi, RNA was isolated from the cultures and analyzed using the Affymetrix Mouse 430 2.0 chip. Genes whose expression increased at least 2-fold during type II:*MAF1* infection relative to type II and were statistically significant at an FDR <5% for differential expression using SAM [67] are depicted. Colors indicate the deviation of each gene's signal above (yellow) and below (blue) its mean expression value across all six samples. (D) Primary mBMDMs were infected with type II, type II:*MAF1* strains, or mock-infected. Supernatants were collected at 17 hpi, passed through a 5 µm filter, and stored at −80°C. Samples were thawed on ice immediately prior to processing on the Luminex mouse 26plex (Millipore, USA) platform. Data shown are for the four cytokines with a MAF1-dependent difference and from three biological replicates (different days) ± s.e.m. Asterisks denote significant differences between mBMDM cells infected with type II versus type II:*MAF1*, *p<0.05, ***p<0.001, ****p<0.0001 using a two-factor ANOVA analysis.

As previously mentioned, HMA occurs during infection with the vacuolar pathogens *C. psittaci* and *L. pneumophila*. To address the consideration that HMA represents a conserved host response to vacuolar pathogens and might be the result of toll-like receptor (TLR) or IFN-dependent signaling, we infected bone marrow isolated from MyD88^−/−^, IFNAR^−/−^, and wild-type mice with type I and type II parasites (MyD88 is a common part of the signaling pathway for many TLRs, and IFNAR is the shared receptor used by type I IFNs). Electron microscopy showed no differences in HMA in these mutant bone marrow cells relative to wild-type cells (unpublished data). Given our growing understanding of how different *Toxoplasma* strains vary in their interaction with the host, we hypothesized that HMA might be involved in the strain-specific ability of *Toxoplasma* to alter innate immune signaling in infected host cells. Interestingly, early observations of HMA described the presence of “giant mitochondria” at the *Toxoplasma* PVM during infection [35], and more recently, the proteins that regulate mitochondrial morphology have been found to play a crucial role in modulating cellular immune signaling during viral infection [36]–[38]. To determine if HMA results in morphological differences in the associated host mitochondria, the average sizes of unassociated (cytosolic) mitochondria and PVM-associated mitochondria in mouse bone-marrow-derived macrophages (mBMDMs) infected with type I parasites were measured in electron micrographs. The analysis was also done during type II:*MAF1* infection for confirmation. The results showed that in mBMDMs infected with type I or type II:*MAF1* parasites, the average cross-sectional area of a PVM-associated mitochondrion is approximately 3-fold greater than the average for cytosolic mitochondrion (Figure 6A and B). These changes in morphology are consistent with the elongation of mitochondria observed during viral infection [37] and support a general role for alterations in mitochondrial morphology having an impact in microbial infection.

Previous work has shown that signaling through the mitochondrion can alter transcription of proinflammatory cytokines [39]. To investigate the role of HMA in the immune response to *Toxoplasma*, microarrays were used to compare the transcriptional profiles of MEFs 8 hpi with type II and type II:*MAF1* parasites. The microarray analysis revealed that the expression of several chemokines and IFN-gamma-induced genes differed significantly in a MAF1-dependent manner (Figure 6C) and that three of the greatest transcriptional changes were seen for the proinflammatory cytokines CXCL2, CSF2, and IL6, with respective increases in expression in the type II:*MAF1*–infected cells of ∼4.6-, 4.8-, and 5-fold. Using Gene Set Enrichment Analysis (GSEA), we found that canonical immune pathways, including JAK-STAT, NFAT, and IL10, were among the most significantly enriched gene sets up-regulated during type II:*MAF1* infection relative to type II wild type (Figure S7). Although we cannot exclude the possibility that MAF1 mediates these effects independent of its role in HMA, these data suggest that HMA in type II parasites has a significant impact on the host cell's transcriptional response to *Toxoplasma*, especially in terms of innate immune signaling.

To explore this issue further, levels of 26 cytokines, chemokines, and growth factors were measured from supernatants of mBMDMs 3 hpi and 17 hpi with type II and type II:*MAF1* parasites using the Luminex Mouse 26-plex (Meso Scale Discovery, services rendered by Stanford Human Immune Monitoring Center). mBMDMs were used because monocytes have been shown to account for approximately 60% of infected cells during a peritoneal infection with *Toxoplasma* and are required for immunity to this parasite [40]. At 3 hpi, no significant differences were seen between type II and type II:*MAF1*–infected mBMDMs (unpublished data), whereas at 17 hpi, we observed increased secretion of IL6 and IL10 and the proinflammatory chemokines RANTES (CCL5) and the murine IL-8 homologue KC (CXCL1) in cells infected with type II:*MAF1* (Figure 6D). Together, these results argue for a key role for HMA in modulation of the innate immune response during infection.

### Loss of HMA Is Associated with Serum Cytokine Changes *in Vivo*

We and others have previously shown that type I parasites generate a profoundly different immunological response compared with type II parasites, which activate NF-κB and result in increased IL-12 production [14],[15],[41],[42]. To avoid the possibility that the impact of type II on other immune pathways might interfere with our interpretation of the results, we addressed whether the loss of HMA in its natural context (type I background) impacted infection *in vitro and in vivo*. To test this, WT MEFs were infected with type I and type I:*Δmaf1* parasites, and at 8 hpi, the RNA was isolated and processed for microarray analysis. Interestingly, unlike the 231 genes differentially regulated between type II:*MAF1* and type II infection, only eight genes were observed to be significantly induced in a MAF1-dependent manner during type I infection, including SerpinB2 and IL-11 (Figure S8); the increased expression of SerpinB2 and IL-11 receptor alpha (IL-11rap) was also MAF1-dependent during type II:*MAF1* infection (Figure 6C) and might represent part of a HMA-induced signaling pathway in host cells.

**Figure 7:**
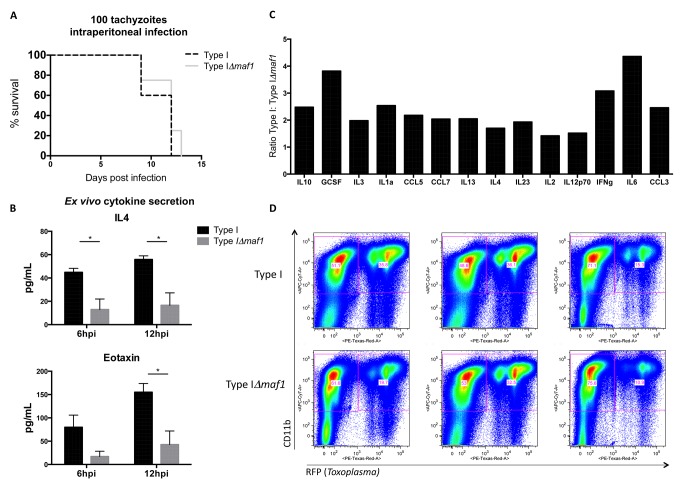
Loss of HMA is associated with serum cytokine changes *in vivo*. (A) C57BL/6 mice (*n* = 5 per parasite strain) were infected subcutaneously with 100 type I or type I:*Δmaf1* tachyzoites and time to death assessed. (B) Mice (*n* = 4) were infected intraperitoneally with 100 tachyzoites of a type I or a type I:*Δmaf1* strain. At 5 dpi, PECs were isolated and supernatants analyzed for cytokine content at 6 and 12 h postisolation. (C) Sera (*n* = 12 per parasite strain) and (D) PECs (*n* = 3 per parasite strain) were isolated from mice infected intraperitoneally with 100 tachyzoites of a type I:*RFP*+ or type I:*Δmaf1:RFP+* strain. Values represent the ratio of the average MFI per cytokine in sera of type I/type I:*Δmaf1–*infected mice. Results from three independent experiments were pooled and values reported if *p*<0.05 using an unpaired *t* test to compare average MFI values. PECs were permeabilized and labeled with APC/Cy7-conjugated CD11b antibody and processed for FACS analysis.

To address whether the loss of HMA in the type I background impacted virulence in the murine host, we compared lethality following intraperitoneal infection of C57/BL6 mice with type I and type I:*Δmaf1* tachyzoites and observed no differences (Figure 7A). This was expected, as previous analyses of the same II×III F_1_ progeny used to map HMA, as well as crosses of type I with types II and III, showed no indication of a significant mouse-virulence trait on chromosome II, the MAF1-encoding chromosome [43]–[45]. Although our results confirm that *MAF1* is not a major virulence determinant using the crude measure of mortality by intraperitoneal infection in mice, they did not address the possibility of other differences in the host response to infection by type I and type I:*Δmaf1* parasites. To test for such effects, PECs were isolated from mice 5 dpi with type I or type I:*Δmaf1* tachyzoites and their cytokine production assessed on the Luminex platform at 6 h and 12 h postisolation. The results showed differences (*p*<0.05) in two cytokines, IL4 and eotaxin, and interestingly, both were present at lower levels in cultured supernatant of PECs from the type I:*Δmaf1*–infected mice compared to mice infected with type I (Figure 7B) parasites. To test the effect on the animal as a whole, sera from similarly infected mice were also analyzed 5 dpi. We observed that, as with IL4 and eotaxin secretion from isolated PECs, 14 of the 26 cytokines examined were present at lower levels in mice infected with type I:*Δmaf1* relative to type I parasites (Figure 7C). These differences included IL6, IL10, and RANTES (CCL5), which were increased in a MAF1-dependent manner during type II versus type II:*MAF1* infections in mBMDMs (Figure 5D). To determine if the differential cytokine production observed was a result of differences in parasite burden, mice were infected with RFP-expressing type I and type I:*Δmaf1* tachyzoites. At 5 dpi, the same time point used for the sera analyses, PECs were isolated and analyzed for RFP expression using flow cytometry and shown to exhibit comparable parasite levels (Figure 7D). Thus, although the *MAF1* genotype appears to have no effect on lethality or parasite burden during the acute stages of an i.p. infection, these results suggest some role for MAF1 in modulating the immune response and support for the notion that HMA is a novel strategy by which an intracellular pathogen can interfere with host signaling.

## Discussion

We have identified the parasite effector that mediates strain-specific HMA during infection with certain types of *T. gondii* and have shown that expression of this effector also impacts the host immune response. Our findings suggest a new dimension to the recently discovered role for mitochondria in the innate immune response [46] and suggest a novel approach by which a microbe may manipulate host cell processes, be they metabolic or immune in nature.

### Recruitment of Host Mitochondria Is Strain-Specific

It is well known that the three archetypal *T. gondii* strains that dominate in Europe and North America differ in several important host interactions including the ability to inactivate mouse immunity-related GTPases (IRGs) that otherwise attack the PVM [47] and to phosphorylate host signal-transducers and activators of transcription (STATs) [48],[49]. We show that HMA is yet another dramatic way in which the three dominant genotypes differ and so is part of a complicated calculus in the parasite's evolution with the optimal solution (HMA^+^ or HMA^−^) likely depending on the interplay with other factors. For example, it could be that there are strain-specific differences in the need for host metabolites produced by the mitochondria (e.g., lipoic acid [19]) and/or susceptibility to the damage mitochondrial metabolites can cause (e.g., reactive oxygen species [50]). Of note, type II strains but not types I and III encode a version of the soluble factor, GRA15, that stimulates NF-κB production in infected cells [14]. It may be that a combination of HMA and NF-κB stimulation would be suboptimal to the parasite compared to either on its own, but the extraordinary success of type II in certain regions of the world clearly indicates that this strain is well optimized for at least some niche. It is also possible that the function served by HMA in types I and III is provided by a completely different mechanism in type II, but until that function is known, this is difficult to speculate about.

Most models predict that different traits evolved in certain *Toxoplasma* strains because of their interaction with a particular host species or host state. IRGs, for example, are crucial parts of the immune armamentarium in mice but are functionally lacking in primates, possibly explaining the polymorphisms seen in the *Toxoplasma* effectors, ROP5 and ROP18, that neutralize them. MAF1, like ROP5 or ROP18, may have evolved as a crucial defense to the immune response in a specific host. We note that the dramatic difference in murine fibroblast transcription observed in type II:*MAF1* versus type II infection was not observed in type I versus type I:*Δmaf1* infection. This is likely due to the very different host responses seen in cells infected with type I versus type II parasites. We saw no difference in HMA in the three species of host cells examined (human, mouse, and dog), but we cannot exclude that some untested host exists where type II tachyzoites do show HMA, although this would likely require host-specific activation of transcription from the *MAF1* locus given that *MAF1* expression in type II parasites is essentially quiescent when grown *in vitro*.

The genes responsible for the HMA seen during *Legionella* and *Chlamydia* infection have not yet been identified, but no *MAF1* homologue is seen in their respective genomes by BLAST analysis (unpublished data). Interestingly, HMA is not seen with all species of these two genera: *L. pneumophila* and *C. psittaci* induce the phenomenon, but *Legionella micdadei* and *C. trachomatis* do not [9],[51], suggesting the presence of a particular niche occupied by the HMA-inducing species of these bacteria where the phenomenon is advantageous. Although it seems likely that HMA is an example of convergent evolution in these bacteria and *Toxoplasma*, the biological implications of HMA in these bacteria will be difficult to dissect until HMA^+^ and HMA^−^ versions are engineered in the same genetic background.

### MAF1 Is a Novel Example of a Microbial Factor That Recruits Host Organelles

Although these results demonstrate that MAF1 directly binds host mitochondria and is crucial for HMA, they do not make any prediction as to the mitochondrial binding partner and whether HMA is directly responsible for the observed alterations in the morphology of the associated mitochondria. No other molecule has been described with the ability to mediate HMA, and the sequence of MAF1 did not provide any obvious clues to mechanism. Previous models involving ROP2 as the mediator of HMA proposed that a mitochondrial import signal on the protein's N-terminus is recognized by the import machinery on the mitochondria, and an incomplete, stalled uptake of the protein results in HMA. This exact mechanism seems unlikely given that the MAF1 C-terminus appears to play a key role, as seen by the inability of the MAF1_CHA construct to mediate HMA, but our results would be consistent with a model involving uptake of C-terminal import signals as shown for the yeast DNA helicase Hmi1P [52]. Further understanding of the mechanism by which MAF1 mediates HMA will require identification of the host molecule(s) bound by MAF1.

### How Does HMA Result in Changes in the Host Immune Response?

Mitochondria act as a nexus for a wide range of host cellular processes and signaling networks including chemotaxis, calcium signaling, apoptosis, and immune signaling. It has been previously reported that during *Toxoplasma* infection, over one-third of all modulated host proteins are mitochondrial [53],[54], a significant enrichment considering mitochondrial proteins are predicted to account for only 4.25% of the cell proteome [54]. This is consistent with HMA having a profound effect on mitochondria during a *Toxoplasma* infection, similar to what has been reported during bacterial and viral infections where substantial changes are observed in mitochondrial morphology and outer mitochondrial membrane composition [50],[55]. Our results on the increased cross-sectional area of PVM-associated mitochondria also suggest HMA affects their physiology, although we have not directly assessed this in our experiments here.

The identification of the key protein mediating HMA allowed us to show that this phenomenon is associated with a significant alteration of transcription of host cell immune genes. We do not know the mechanism by which HMA results in the transcriptional responses or cytokine responses observed, or whether it is directly responsible for these changes. It is well known, however, that during viral infection, signaling through MAVS results in recruitment of cytosolic factors to the outer mitochondrial membrane; activation of different transcription factors including NF-kB, IRF3, and IRF7; and subsequent production of type I IFNs and inflammatory cytokines [39]. Although there was a significant enrichment for genes with NF-kB binding sites in their promoters by Transcription Binding Site analysis of the microarray data during type II:*MAF1* infection of mBMDMs (unpublished data), there was no evidence for IRF3 or IRF7 activation. Interestingly, although the absence of MAVS did not appear to affect HMA in type I–infected cells, five of the eight genes whose induction or repression was MAF1-dependent in MEFs during type I infection were also MAVS-dependent (Figure S8). These include SerpinB2 and TSG14 (tumor necrosis factor–inducible gene 14) but not, however, IL11, suggesting MAVS may play some role in MAF1-induced immune changes in the host cell.

Although we cannot exclude the possibility that MAF1 is mediating the observed transcriptional and immune responses independently of HMA, we hypothesize that type I and III *Toxoplasma* co-opt mitochondrial signaling using HMA to generate a cytokine milieu conducive to parasite survival and efficient dissemination. Interestingly, the cytokines whose levels were increased in HMA^+^ strains during mBMDM infection *in vitro* and in PECS *ex vivo*, including IL6, CCL5, and IL10, are not those that would be expected to change based on interference with known mitochondrial signaling pathways (i.e., MAVS, NF-kB, IRF3/7 [22],[23]). These data suggest that HMA might be functioning through a yet unidentified pathway linking mitochondria and innate immune signaling, further highlighting the connection between mitochondria and immunity. It is possible that the observed changes in mitochondrial morphology reflect this manipulation, as it has previously been shown that the machinery that regulates mitochondrial fission and fusion (e.g., Mitofusin 1 and 2) are also necessary in the signaling cascades that result during viral infection [37],[38]. Future work will be required to map out the pathways that link HMA to the observed differences in cytokine responses, as well as to determine how the consequences of HMA impact the host–parasite interaction. Our identification of strain-specific differences in HMA during infection with *Toxoplasma* and the molecular basis underlying those differences provides a model that can be used to dissect the biological role of HMA and the possibly novel ways by which mitochondria engage immune-signaling pathways.

## Materials and Methods

### Parasite and Cell Culture

*T. gondii* parasites of the type I (RH*Δhxgprt*), type II (ME49*Δhxgprt*), and type III (CEP*Δhxgprt*) strains [deleted for the *hypoxanthine–xanthine–guanine phosphoribosyl transferase* (*HXGPRT*) gene] were maintained by serial passage in HFF monolayers. The GFP-expressing strain of RH (RHgfpluc) [56] and the F1 recombinant progeny derived from type II (ME49) and type III (CEP) [57] crosses used for the co-infections have all been described previously.

mBMDMs were obtained from female C57BL/6 mice as previously described [14] and cultured in 20% M-CSF, 10% heat-inactivated FBS, 2 mM L-glutamine, 1 mM sodium pyruvate, 1× DMEM (Dulbecco's modified Eagle's medium; Invitrogen) plus nonessential amino acids, and 50 µg/ml each of penicillin and streptomycin. MEFs (a gift from Z.J. Chen, University of Texas Southwestern Medical Center at Dallas) and HFFs were grown in complete DMEM (cDMEM) supplemented with 2 mM glutamine, 100 U/ml penicillin, and 100 µg/ml streptomycin and 10% heat-inactivated fetal calf serum (FCS).

### Cloning MAF1 Paralogs from Type I and Type II Strain Parasites

Genomic DNA from either RH88 (type I) or ME49 (type II) was used as a template in PCR reactions using Platinum Taq HiFi polymerase (Invitrogen; Life Technologies) with primers 5′ AGGGATACGAACAACTCGCTTAT 3′ (forward) and 5′ GTCCAGCATGCTAGCCAGATACGT 3′ (reverse). PCR was performed under standard conditions, except extension times for each cycle were carried out for 7 min at 68°C to minimize chimera formation between different MAF1 copies. PCR products were gel-purified and cloned into the PCR2.1 Topo vector and completely sequenced. Inserts ranged in size from 2,995 to 3,119 bp and were spliced *in silico* based on the annotated splice sites for TGGT1_053770. Sequences were aligned using ClustalW (http://www.ebi.ac.uk/Tools/msa/clustalw2/) and visualized using Jalview.

### Generation of Transgenic Parasites

For generation of an N-terminally hemagglutinin (HA)-tagged MAF1 expression construct (pMAF1), the promoter (∼1.5 kb sequence upstream of the start codon) was cloned into the pGra-HA_HPT vector [58] at the HindIII and NsiI sites using forward primer CCAAGCTTCTGCGACGTGATCGTGGCAA and reverse primer CCATGCAT**CGCGTAGTCCGGGACGTCGTACGGGTA**ACCGGCGGTCAG [reverse primer encodes an HA tag (bolded) fused immediately downstream of the region encoding the predicted signal peptide]. The coding region downstream of the signal peptide of MAF1 up to the stop codon was cloned into the NsiI and PacI sites with forward primer CCATGCAT**GGTGGT**CTAGGCAGTCAGATGTCGG [introduces two glycine residues (bolded) after the signal peptide] and reverse primer CCTTAATTAATC AGTCCAGCATGCTAGCCAG. Transgenic parasite strains were made by electroporation of the parental type I and II *T. gondii* strains with 20 µg of linearized pMAF1 and applying mycophenolic acid and xanthine selection to isolate and clonally expand parasites positive for HXGPRT expression [59]. Type I strains transfected with pMAF1 are referred to as type I:TGGT1_053770 or type I:*MAF1*; type II strains transfected with pMAF1 are referred to as type II:TGGT1_053770 or type II:*MAF1*.

A construct for knocking out the endogenous *MAF1* locus (pMAF1LKO) was created from the parental pTKO vector [60]. Briefly, an ∼850 bp region of genomic DNA corresponding to sequences upstream of the TGGT1_053770 start codon (5′ homology region or 5′ HR) and a ∼720 bp region of genomic DNA corresponding to sequences upstream of TGGT1_ 064860 (3′ HR) were cloned into NotI/EcoRV and HpaI/ApaI restriction sites that flank the *T. gondii HXGPRT* gene and *GRA2* 3′-UTR in the pTKO vector, respectively. Primers for amplification of the 5′ insert were AAGCGGCCGCCGAGACAGAGAGCAGTGCCAA and AAGATATCGGATGTGGCTCCACTGGTGAAT; for the 3′ insert, they were CCGTTAACGTGAGAGCTCCATCATCGACTCCTT and AAGGGCCCGGTGTGCCCGCTTGGATCAA. The type I:*Δmaf1* parasite strain was made by electroporating type I (*ΔhxgprtΔku80*) parasites with 25 µg of linearized pMAF1KO and selecting for HXGPRT-positive parasites as previously described [30].

### Immunofluorescence Assay

HFFs were grown to confluency in 24-well plates. For staining with MitoTracker (Invitrogen), medium was replaced with prewarmed DMEM containing MitoTracker at a concentration of 50 nM. After 30 min of incubation at 37°C, cells were washed and then infected with parasites in prewarmed cDMEM. At 4 hpi, cells were washed in PBS and fixed in prewarmed cDMEM containing 3.7% formaldehyde for 15 min. Otherwise, parasites were allowed to invade confluent HFF monolayers on coverslips for 6 and 12 h, fixed in 4% formaldehyde, permeabilized with 0.1% Triton-X100 for 20 min, and blocked in PBS supplemented with 3% BSA. Coverslips were then incubated with 3F10 (anti-HA) antibody (Roche, Palo Alto, CA), antibodies specific for TOM20 (SCBT, Santa Cruz, CA), or mouse polyclonal sera to MAF1 for 1–3 h at room temperature (RT). Fluorescent secondary antibodies (Invitrogen/Molecular Probes, Carlsbad, CA) were used for antigen visualization. Coverslips were mounted in VectaShield (Vector). Phase and fluorescence images were captured with a Hamamatsu Orca100 CCD on an Olympus BX60 (100×) and FV1000 Olympus confocal scope (DIC) and processed using Image-Pro Plus 2.0 (MediaCybernetics).

### Western Blot Analysis

For MAF1 immunoblotting, cells were lysed in sodium dodecylsulfate sample buffer, and samples were resolved on 8% SDS-polyacrylamide gels and blotted to PVDF membranes (Millipore, Billerica, MA). Following gel transfer, membranes were blocked with PBS-0.05% Tween 20 (PBS-T) and 0.05% nonfat dry milk for an hour and then incubated in mouse anti-MAF1 sera at 1∶250 for 3 h. Following incubation, blots were washed three times in PBS-T and then incubated with horseradish peroxidase (HRP)-conjugated anti-mouse antibodies at a 1∶2,500 dilution. The membrane was stripped (Invitrogen stripping buffer) and reprobed with rabbit anti-SAG1 antibody at a 1∶100,000 dilution in blocking solution for 1 h. After three PBS-T washes, the blot was incubated with HRP-conjugated anti-rabbit antibodies at a 1∶3,000 dilution and developed using a chemiluminescence system (Pierce Chemical Co., Rockford, IL). For assessing phosphorylation, lysates from extracellular type I parasites and type I–infected HFFs (intracellular) were treated with and without calf intestinal phosphatase (CIP) for 1 h and loaded in each lane. The membrane was probed with anti-HA antibody conjugated to peroxidase and developed as stated above.

### Antibody Generation

Recombinant MAF1 protein (amino acids 134–443) was expressed with a C-term His-6× tag as previously described [61] and was a gift of M. Tonkin and M. Boulanger (University of Victoria). Blood of naïve female Balb/c mice was drawn and tested against recombinant MAF1_I_ to establish baseline reactivity. Each mouse was then immunized intraperitoneally (IP) with 0.1 mg of MAF1 antigen in a 1∶1 mixture with RIBI (Corexia) in a final volume of 200 ml. Three IP injections of 50 mg in a 1∶1 mixture with RIBI (final volume 200 ml) were administered on day 14, 35, and 56. The final bleed was taken at week 13; collected sera were verified by IFA and Western blot analysis.

### Electron Microscopy

Monolayers of BMMs grown on glass coverslips were synchronously infected with parasites as described above and 6 h later fixed in Karmovsky's fixative—2% glutaraldehyde and 4% paraformaldehyde in 0.1 M sodium cacodylate pH 7.4—for 1 h at RT. Following this, the cultures were stained with 1% osmium tetroxide for 1 h at RT, washed 3× with ultrafiltered water, then stained in 1% uranyl acetate overnight. After a series of ethanol washes and propylene oxide incubations, the samples were placed into molds with labels and fresh 100% EMbed-812 resin in a 65°C oven overnight.

Sections were taken between 75 and 90 nm, picked up on formvar/Carbon coated 75 mesh Cu grids, and stained for 20 s in 1∶1 saturated uranyl acetate (7.7%) in acetone followed by staining in 0.2% lead citrate for 3 to 4 min. Samples were observed in the JEOL 1400 TEM at 80 kV, and photos were taken using a Gatan Multiscan 791 digital camera.

For quantification of HMA, 30 images were taken of PVs in infected cells and 20 (or as indicated) were randomly selected for further analysis by ImageJ. Image J was used to measure the percentage of the PVM perimeter closely associated with host mitochondria (PVM associated with mitochondria/total PVM×100).

### Sequence Read Alignments

Raw sequence reads for *T. gondii* (strains GT1, ME49, and VEG) were downloaded from the NCBI trace archive in FASTA format. Reads were aligned to the *T. gondii* ME49 genome (version 7.0; ToxoDB) using BLAT with the following parameters: -fastMap -minIdentity = 95 -minScore = 90 [62]. Following conversion of the blat output file (psl format) to the SAM format using the psl2sam.pl script within the Blat distribution, the SAM file was converted to a sorted BAM file using Samtools [63]. Sequence coverage was determined in each 500 bp window using coverageBed, distributed with BEDtools [64]. Output was uploaded in R to generate sequence coverage plots of the expanded locus encompassing *TGGT1_053770* plus 20 Kb of upstream and downstream sequence. The average read coverage per 500 bp window in the 20 Kb sequence upstream of the expanded locus was used to normalize the coverage plots for all three strains.

### Retroviral Expression

For generation of MAF1 expression in MEFs, the coding region for the full-length MAF1 was PCR-amplified from pMAF1 using the forward primer TTC TCG AGC ACC ATG GCC GGT TAC CCG TAC GAC G and reverse primer TTG CGG CCG CTC AGT CCA GCA TGC TAG CCA G and cloned into the XhoI and NotI sites of the MSCV2.2 vector (gift from the Barton Lab, University of California, Berkeley) downstream of the CMV promoter. The forward primers included the Kozak consensus sequence (CACC) before the start codon. The MAF1 transgene starts at the signal peptide cleavage site (amino acid 24), has an N-terminal HA tag after the signal peptide cleavage site, and runs through to the stop codon. Phoenix cells were transfected with 15 µg of pMSCV_MAF1 using Lipofectamine LTX (Invitrogen). About 24 hpi transfection, the supernatant was removed and replaced with fresh cDMEM and the cells transferred to 32°C. The following day the supernatant was filtered with a 22–45 µm filter, polybrene at a concentration of 5 µg/ml was added, and the mix transferred to MEFs at a confluency of 50%. MEFs were incubated for one day at 32°C and then returned to 37°C for expansion. Cells were analyzed for MAF1 expression via immunofluorescence using anti-HA or polyclonal anti-MAF1 antibodies.

### Microarrays

For mouse arrays, WT and MAVS^−/−^ MEFs were grown in a six-well plate to confluency. Parasite strains were syringe-lysed and washed once with PBS. MEFs were infected with type I, type I:*Δmaf1*, type II expressing luciferase (Me49:*LUCΔhxgprt*), or type II:*MAF1* parasites at an MOI of 5 or mock-infected for 8 h after which RNA was isolated using TRIzol (Invitrogen) followed by labeling and hybridization to a mouse Affymetrix array (Mouse 430A 2.0) according to the manufacturer's protocol. Probe intensities were measured with the Affymetrix GeneChip Scanner 3000 7G and were processed into image analysis (.CEL) files with GeneChip Operating Software (Affymetrix). Data were normalized using the Robust Multi-Array Average normalization algorithm. GSEA was used to identify canonical pathways and candidate transcription factors modulated by MAF1 [65],[66]. Statistical significance for differential gene expression was determined using SAM [67]. The delta parameter was adjusted to achieve a false discovery rate (FDR) nearest to 10%, or as indicated, and this delta value was used to select significantly regulated genes. All microarray data have been deposited in the Gene Expression Omnibus (http://www.ncbi.nlm.nih.gov/geo/).

### Bone Marrow Macrophage Secretion

Primary mBMDMs were isolated from female B6 mice and plated to confluency in 24 wells. BMDMs (three wells per condition) were infected with type II (Me49:*LUCΔhxgprt*) or type II:*MAF1* parasites at an MOI of 3 or mock-infected in equal volumes. At 3 hpi and 17 hpi, supernatants from the three wells for each condition were pooled, pushed through a 5 µm filter, and stored at −80°C until analysis. Supernatants were analyzed in the Human Immune Monitoring Core (Stanford, CA) by Luminex Mouse 26-plex kits purchased from Affymetrix and used according to the manufacturer's recommendations with modifications as described below. Briefly, samples were mixed with antibody-linked polystyrene beads on 96-well filter-bottom plates and incubated at RT for 2 h followed by overnight incubation at 4°C. Plates were vacuum-filtered and washed twice with wash buffer, then incubated with biotinylated detection antibody for 2 h at RT. Samples were then filtered and washed twice as above and resuspended in streptavidin-PE. After incubation for 40 min at RT, two additional vacuum washes were performed, and the samples resuspended in Reading Buffer. Each sample was measured in duplicate. Plates were read using a Luminex 200 instrument with a lower bound of 100 beads per sample per cytokine. Data shown are averaged from three independent experiments and are from three biological replicates (different days) ± s.e.m. Asterisks denote significantly different MFIs between mBMDM cells infected with type II versus type II:*MAF1*, * *p*<0.05, *** *p*<0.001, **** *p*<0.0001 using a two-factor ANOVA analysis.

### PEC Isolation

Euthanized C57BL/6 mice (*n* = 4 per parasite strain) were injected with 6 ml cold 1× PBS using a 26 gauge needle. Mice were palpated for 1–2 min, following which fluid contents were aspirated out of the peritoneum. Content was spun down at 1,000 rpm for 5 min, washed, and resuspended in DMEM to a concentration of 2×10^6^ per ml. We incubated 500 µl of this material for 6 or 12 h, passed it through a 0.2 µm filter, and the filtrate was stored at −80°C until Luminex analysis. Samples were analyzed for 26 cytokines using the Luminex platform (see above). Data were reported if *p*<0.05 for an unpaired comparison of the averages.

### Sera Isolation

Terminal bleeds were performed 5 dpi on type I and type I:*Δmaf1–*infected female C57BL/6 (*n* = 9 per parasite strain) and kept at 4°C for 4 h. Bleeds were then spun down at 14,000× rpm, and sera were isolated, aliquoted into fresh tubes, and stored at −80°C until analysis.

### Flow Cytometry

PECs were washed twice with FACS buffer (3% FBS in 1×PBS) and stained for 30 min with surface marker antibody CD11b-APC-Cy7 (BD Biosciences). After staining, the cells were washed with FACS buffer and run on an LSR II flow cytometer (Becton Dickinson). Cells were sorted on a DIVA cell sorter (BD Biosciences) and analyzed using FlowJo software (Tree Star). Cells were measured as a percentage of total intact cells (determined by forward and side scatter measurements). Infected cells were identified by virtue of the red fluorescence of the RFP protein expressed by the engineered *Toxoplasma*.

## Supporting Information

Figure S1 HMA^−^ phenotype is consistent in type II strains. HFFs were labeled with MitoTracker and infected with a type II (Pru) strain. Cells were fixed 4 hpi. Scale bar, 5 µm.
(JPG)

Figure S2 HMA is strain-specific *in vivo*. Mice were infected intraperitoneally with 100 type I tachyzoites or 10,000 type II tachyzoites. At 5 dpi, PECs were isolated and processed for electron microscopy analysis. Transmission electron micrographs of PECs harboring (A) type I and (B) type II vacuoles are depicted. Scale bar, 0.5 µm. (C) Percentage of the PVM associated with mitochondria at type I and II vacuoles as determined by ImageJ analysis of electron micrographs (*n* = 20 for each). Values shown are mean ± SEM. ****p<0.0001 using an unpaired *t* test.
(JPG)

Figure S3 Coinfection with *Toxoplasma* strains that differ in their ability to recruit mitochondria does not alter either strain's HMA phenotype. HFFs were labeled with MitoTracker and co-infected with a type II GFP^+^ strain (green arrowheads) and type I (A) or type III (B) strains. Cells were fixed 4 hpi. Scale bar, 5 µm.
(JPG)

Figure S4 Phenotyping of II×III F_1_ progeny. Genotypes of II×III F_1_ progeny are available at http://toxomap.wustl.edu. Each column represents a different F_1_ as indicated in the top row. Genotypes of progeny at chromosome II markers (one marker per row and as indicated in the left column) are given as type II (red) or type III (yellow). The HMA phenotype of each F_1_ is indicated by a (+) or (−) below each column. The ± for CL11 indicates an ambiguous phenotype. The region in chromosome II implicated by this analysis starts at the left end of chromosome II and ends at marker KT-L379A (denoted by a black box).
(JPG)

Figure S5 Amino acid alignment based on MAF1 gene sequences amplified via long-extension PCR from RH and Me49. The overall amino acid sequence identity between RH_MAF1 and ME49_MAF1 is 94.5%. The predicted signal peptide, TM domain, and proline-rich region are indicated by boxes, and asterisks indicate sites that were found to be phosphorylated [30].
(JPG)

Figure S6 Generation of *MAF1* knockout mutants. (A) Plasmid MAF1LKO, used to transform type I parasites. *HXGPRT* is the selectable marker driven by the *Toxoplasma* dihydrofolate reductase (*DHFR*) promoter; the arrows represent the *MAF1* open reading frames (ORFs), and the parallel lines indicate that the number of *MAF1* copies is unknown. The thick black line represents genomic DNA (gDNA) and the thin black line symbolizes pMAF1LKO. 5′HR and 3′HR represent the 5′- and 3′-homology regions, respectively, used to drive the homologous recombination. Figure not drawn to scale. (B) gDNA from type I and type I:*Δmaf1* parasites was PCR amplified for *MAF1 ORF* and *ROP2 ORF* and analyzed by agarose gel electrophoresis. (C) Lysates from 1×10^6^ type I and type I:*Δmaf1* parasites were loaded in separate lanes. Following polyacrylamide gel electrophoresis and transfer to nitrocellulose, the membrane was probed with polyclonal sera against MAF1 (upper) or SAG1 (lower). (D) Type I and type I:*Δmaf1* parasites were added to HFFs, and cultures were fixed in methanol 6 hpi. Following permeabilization, coverslips were labeled with anti-MAF1_I_ mouse sera followed by incubation with secondary anti-mouse Alexa488 antibodies. Scale bar, 5 µm.
(JPG)

Figure S7 MAF1 alters immune signaling during type II infection. MEFs were infected with type II or type II:*MAF1 Toxoplasma s*trains, or mock-infected. At 8 hpi, RNA was isolated from the cultures, and transcript abundance was determined using the Affymetrix Mouse 430 2.0 chip. Top 5 hits (ranked by normalized enrichment score) shown from GSEA_canonical pathway (c2.cp) analysis.
(JPG)

Figure S8 Role of MAVS in HMA signaling. (A) WT and MAVS^−/−^ MEFs were infected with type I or type I:*Δmaf1 Toxoplasma s*trains at an MOI of 5. At 8 hpi, RNA was isolated from the cultures, and transcript abundance was determined using the Affymetrix Mouse 430 2.0 chip. Genes whose expression was significantly different between type I infection and type I:*Δmaf1* infection in WT MEFs are depicted. Colors indicate the deviation of each gene's signal above (yellow) and below (blue) its mean expression value across all eight samples. (B) Percentage of the PVM associated with mitochondria in WT and MAVS^−/−^ MEFs infected with type I parasites (%HMA) as determined by ImageJ analysis of electron micrographs (*n* = 20 for each). Values shown are mean ± SEM. ****p<0.0001 using an unpaired *t* test.
(JPG)

## Abbreviations

CHA: C-terminus hemagglutinin tag
HA: hemagglutinin tag
HFFs: human foreskin fibroblasts
HMA: host mitochondrial association
hpi: hours postinfection
MAF1: mitochondrial association factor 1
MAVS: mitochondrial antiviral signaling protein
mBMDMs: mouse bone-marrow-derived macrophages
MEFs: mouse embryonic fibroblasts
PECs: peritoneal exudate cells
PVM: parasitophorous vacuole membrane
SEM: standard error of the mean
TOM20: translocase of the outer membrane 20
