# Supplementary figures and images for: Toxoplasma Effector MAF1 Mediates Recruitment of Host Mitochondria and Impacts the Host Response
Source: PLoS Biol. 2014 Apr 29;12(4):e1001845. doi: 10.1371/journal.pbio.1001845 (PMC4004538; doi:10.1371/journal.pbio.1001845)

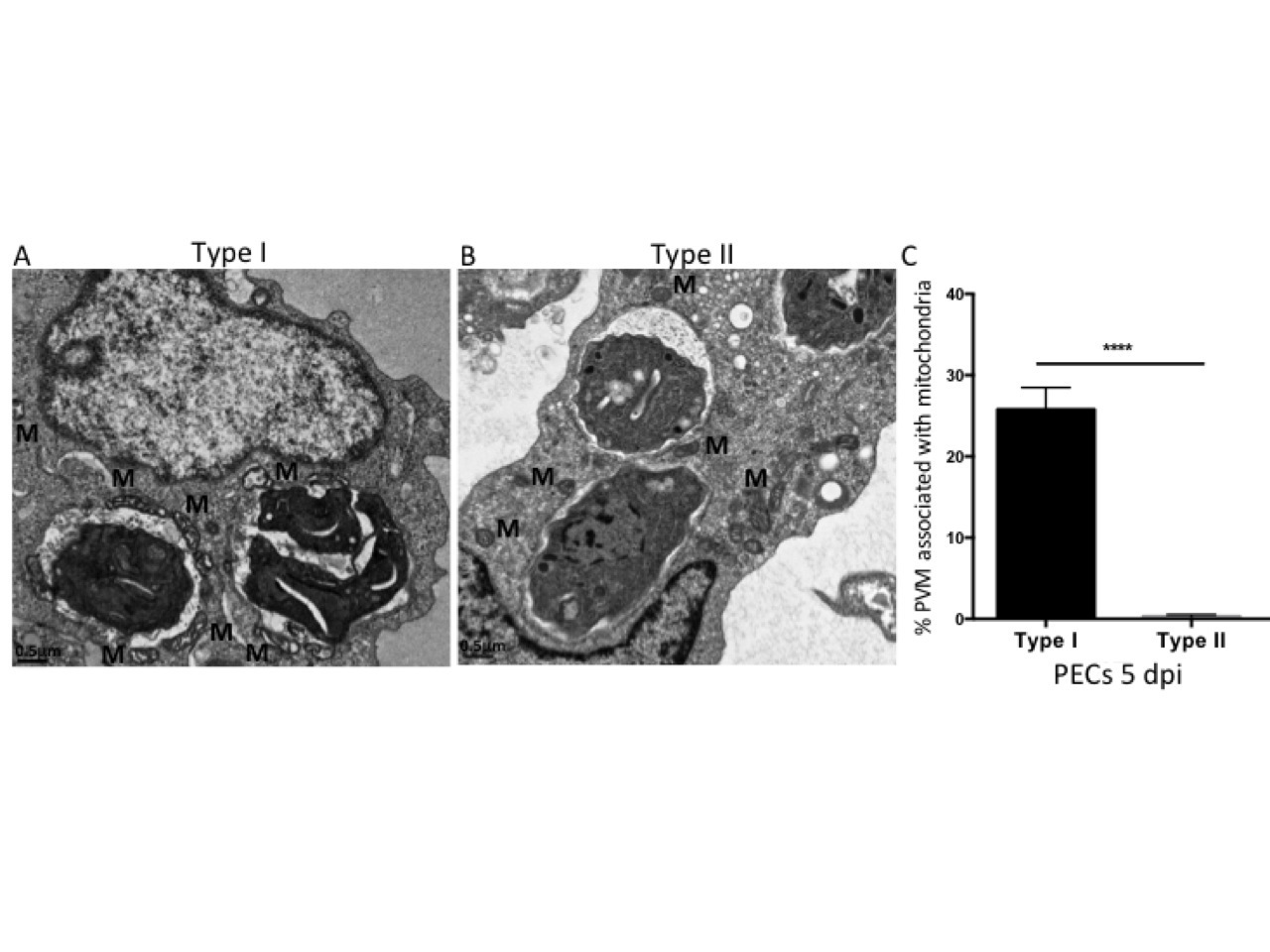

Supplement: Figure S1 — HMA− phenotype is consistent in type II strains. HFFs were labeled with MitoTracker and infected with a type II (Pru) strain. Cells were fixed 4 hpi. Scale bar, 5 µm. (JPG) [file pbio.1001845.s001.jpg]

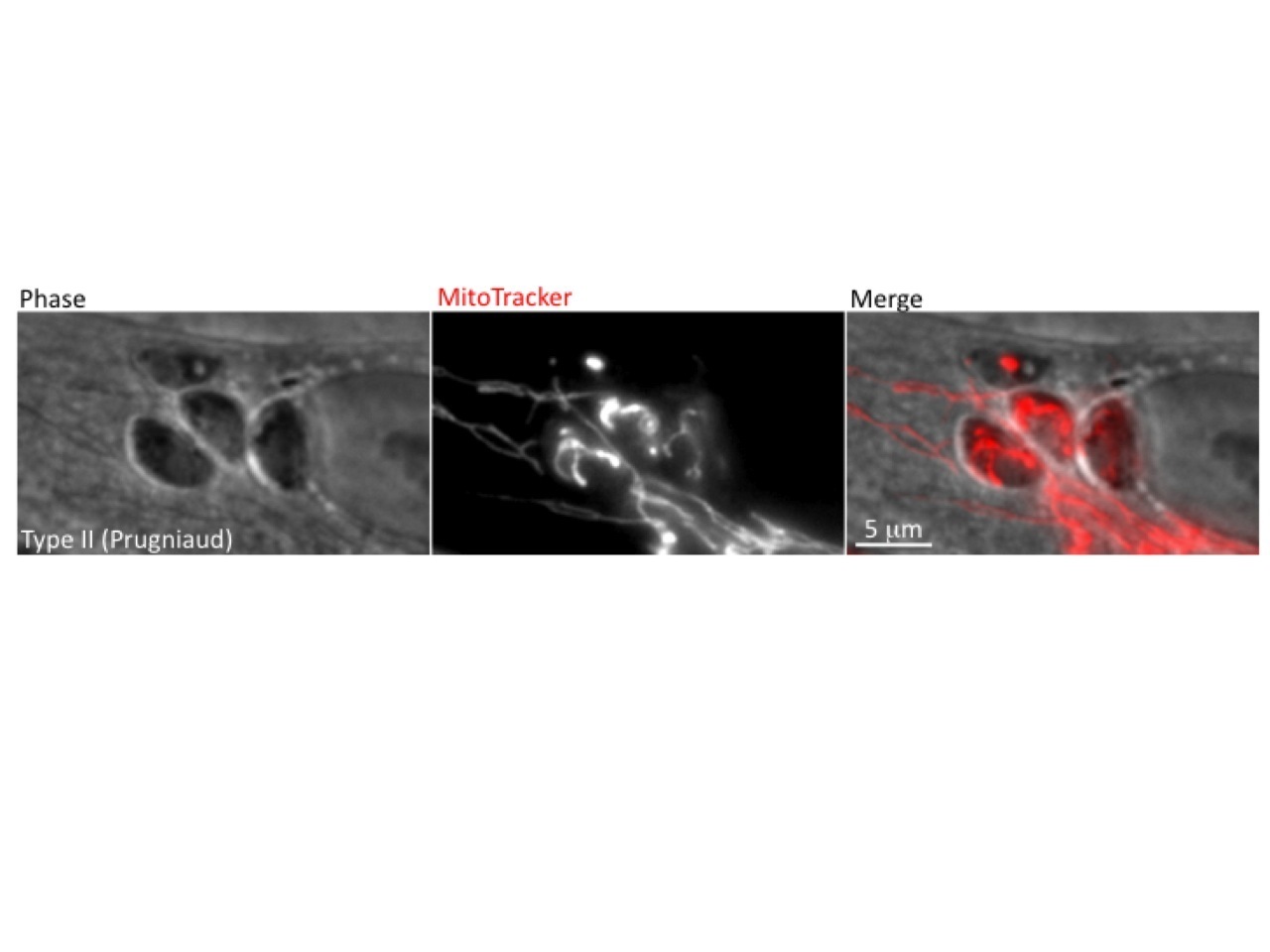

Supplement: Figure S2 — HMA is strain-specific in vivo. Mice were infected intraperitoneally with 100 type I tachyzoites or 10,000 type II tachyzoites. At 5 dpi, PECs were isolated and processed for electron microscopy analysis. Transmission electron micrographs of PECs harboring (A) type I and (B) type II vacuoles are depicted. Scale bar, 0.5 µm. (C) Percentage of the PVM associated with mitochondria at type I and II vacuoles as determined by ImageJ analysis of electron micrographs (n = 20 for each). Values shown are mean ± SEM. ****p<0.0001 using an unpaired t test. (JPG) [file pbio.1001845.s002.jpg]

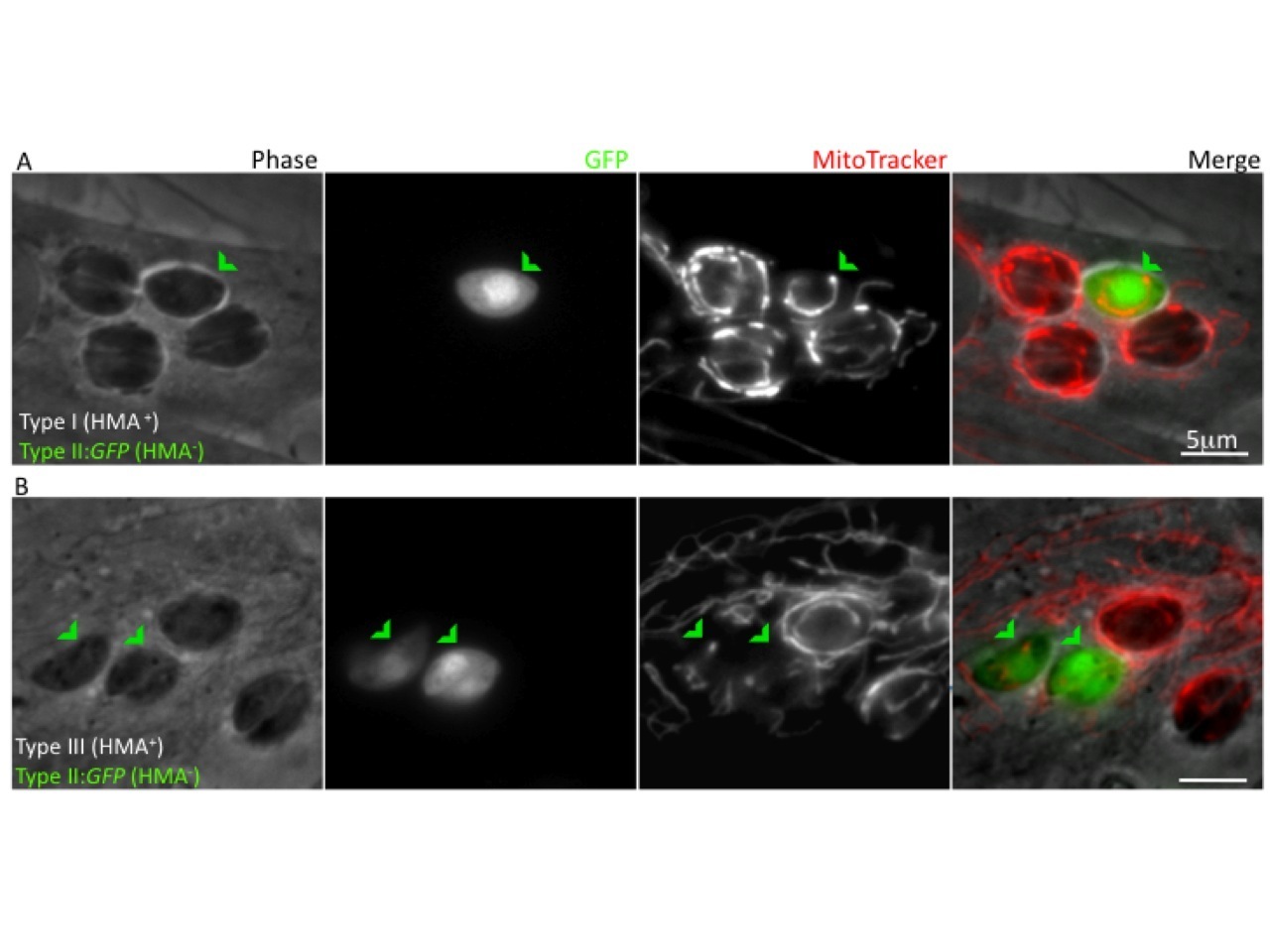

Supplement: Figure S3 — Coinfection with Toxoplasma strains that differ in their ability to recruit mitochondria does not alter either strain's HMA phenotype. HFFs were labeled with MitoTracker and co-infected with a type II GFP+ strain (green arrowheads) and type I (A) or type III (B) strains. Cells were fixed 4 hpi. Scale bar, 5 µm. (JPG) [file pbio.1001845.s003.jpg]

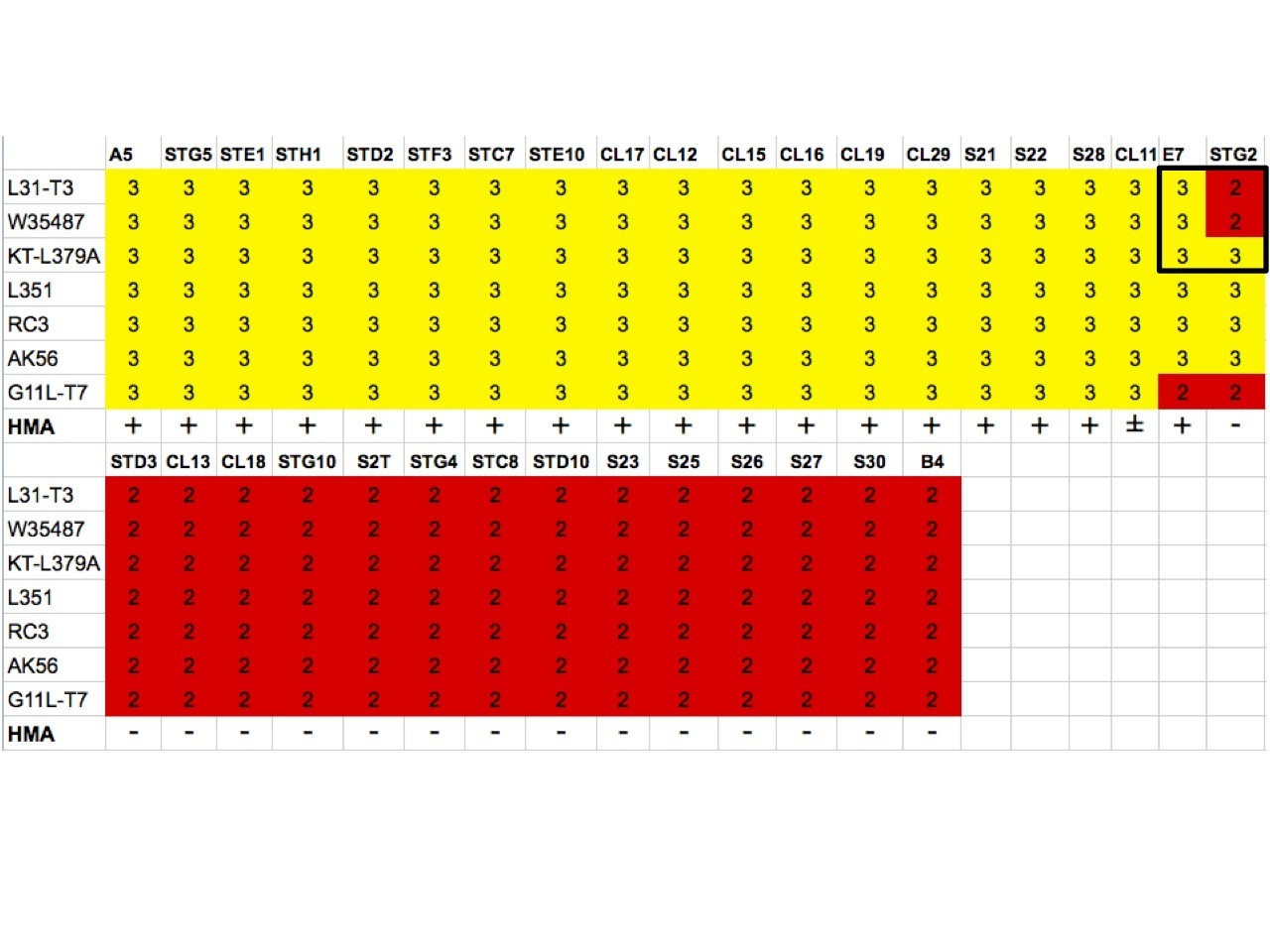

Supplement: Figure S4 — Phenotyping of II×III F1 progeny. Genotypes of II×III F1 progeny are available at http://toxomap.wustl.edu. Each column represents a different F1 as indicated in the top row. Genotypes of progeny at chromosome II markers (one marker per row and as indicated in the left column) are given as type II (red) or type III (yellow). The HMA phenotype of each F1 is indicated by a (+) or (−) below each column. The ± for CL11 indicates an ambiguous phenotype. The region in chromosome II implicated by this analysis starts at the left end of chromosome II and ends at marker KT-L379A (denoted by a black box). (JPG) [file pbio.1001845.s004.jpg]

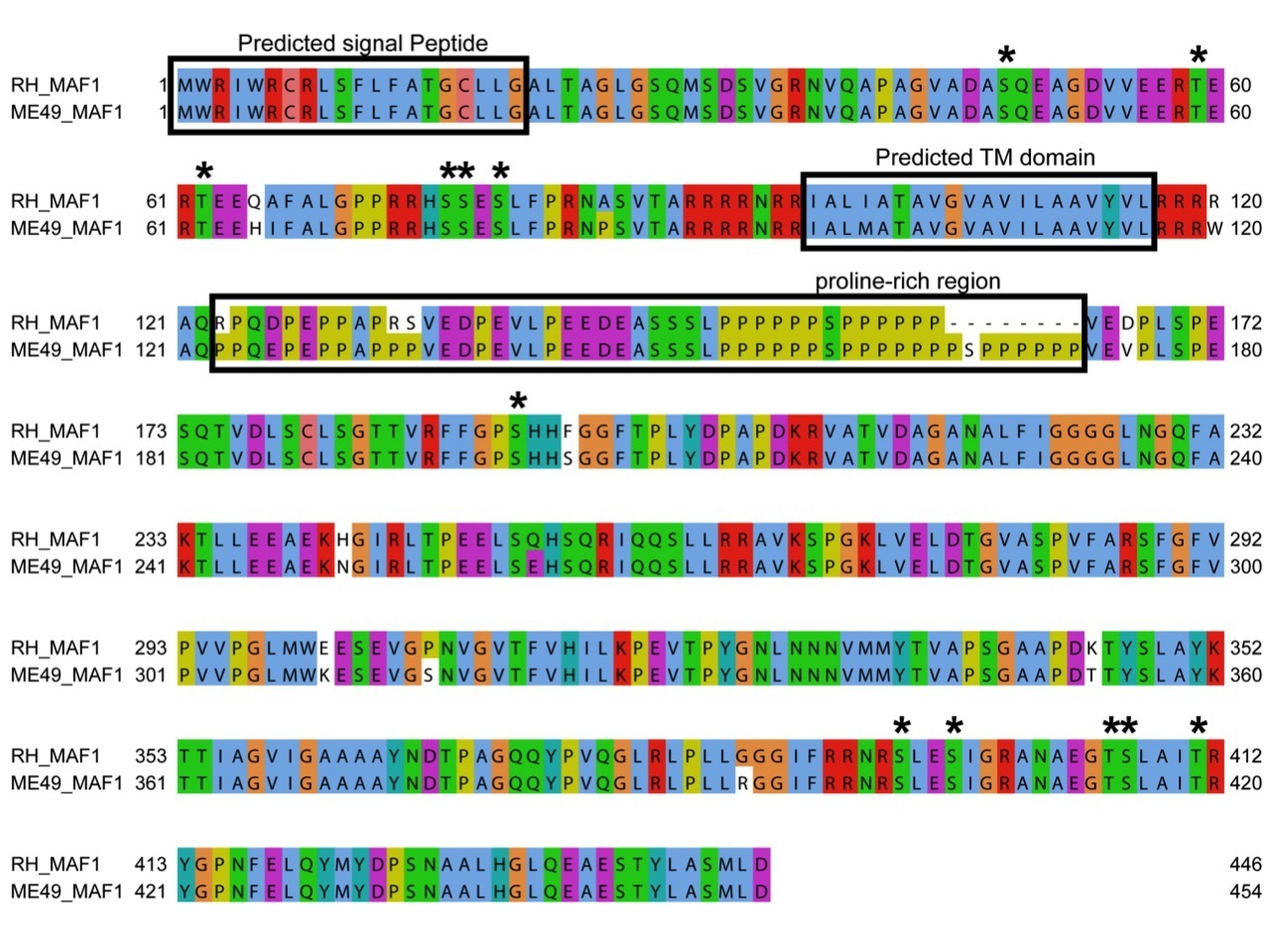

Supplement: Figure S5 — Amino acid alignment based on MAF1 gene sequences amplified via long-extension PCR from RH and Me49. The overall amino acid sequence identity between RH_MAF1 and ME49_MAF1 is 94.5%. The predicted signal peptide, TM domain, and proline-rich region are indicated by boxes, and asterisks indicate sites that were found to be phosphorylated [30]. (JPG) [file pbio.1001845.s005.jpg]

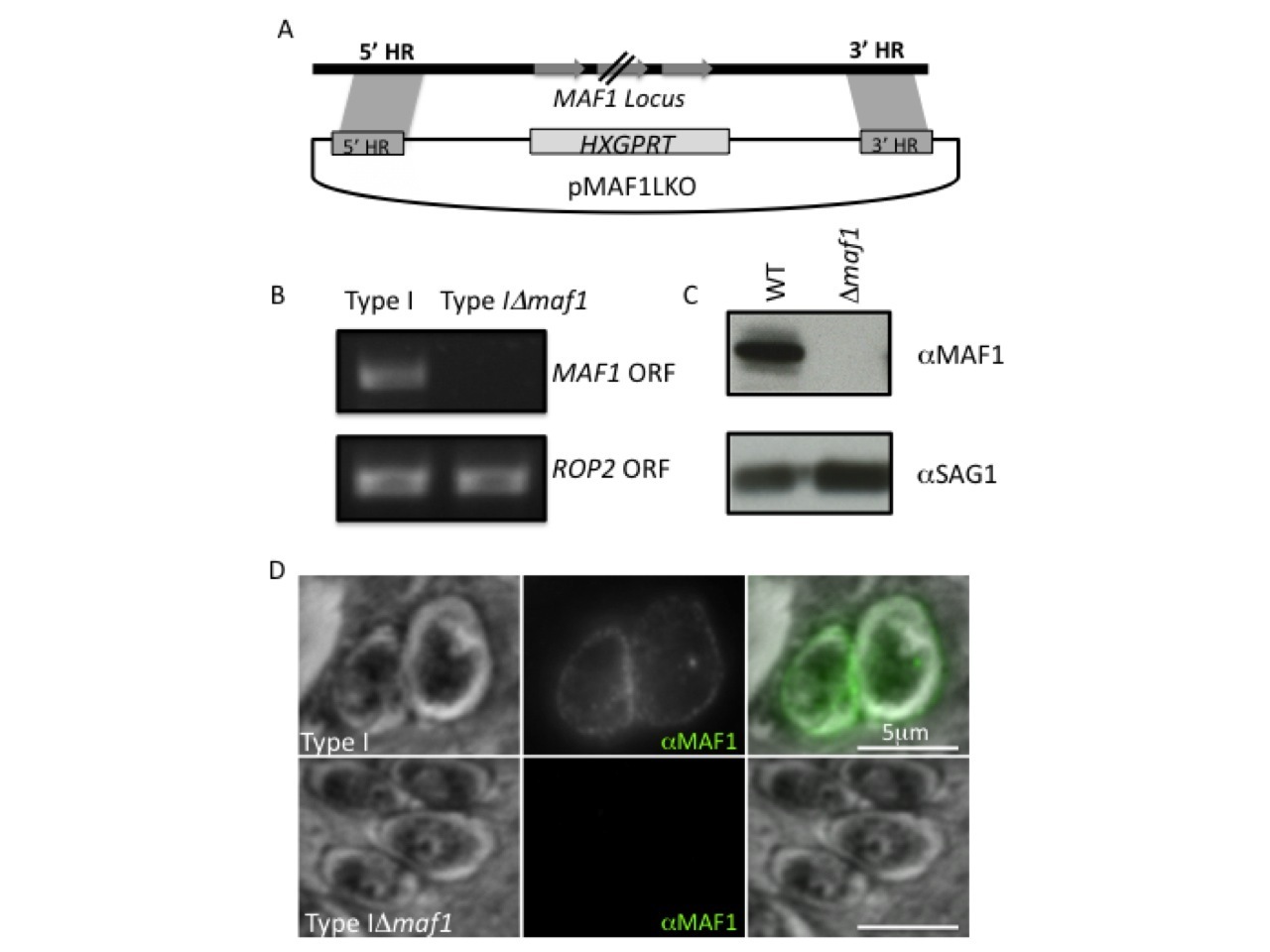

Supplement: Figure S6 — Generation of MAF1 knockout mutants. (A) Plasmid MAF1LKO, used to transform type I parasites. HXGPRT is the selectable marker driven by the Toxoplasma dihydrofolate reductase (DHFR) promoter; the arrows represent the MAF1 open reading frames (ORFs), and the parallel lines indicate that the number of MAF1 copies is unknown. The thick black line represents genomic DNA (gDNA) and the thin black line symbolizes pMAF1LKO. 5′HR and 3′HR represent the 5′- and 3′-homology regions, respectively, used to drive the homologous recombination. Figure not drawn to scale. (B) gDNA from type I and type I:Δmaf1 parasites was PCR amplified for MAF1 ORF and ROP2 ORF and analyzed by agarose gel electrophoresis. (C) Lysates from 1×10^6^ type I and type I:Δmaf1 parasites were loaded in separate lanes. Following polyacrylamide gel electrophoresis and transfer to nitrocellulose, the membrane was probed with polyclonal sera against MAF1 (upper) or SAG1 (lower). (D) Type I and type I:Δmaf1 parasites were added to HFFs, and cultures were fixed in methanol 6 hpi. Following permeabilization, coverslips were labeled with anti-MAF1I mouse sera followed by incubation with secondary anti-mouse Alexa488 antibodies. Scale bar, 5 µm. (JPG) [file pbio.1001845.s006.jpg]

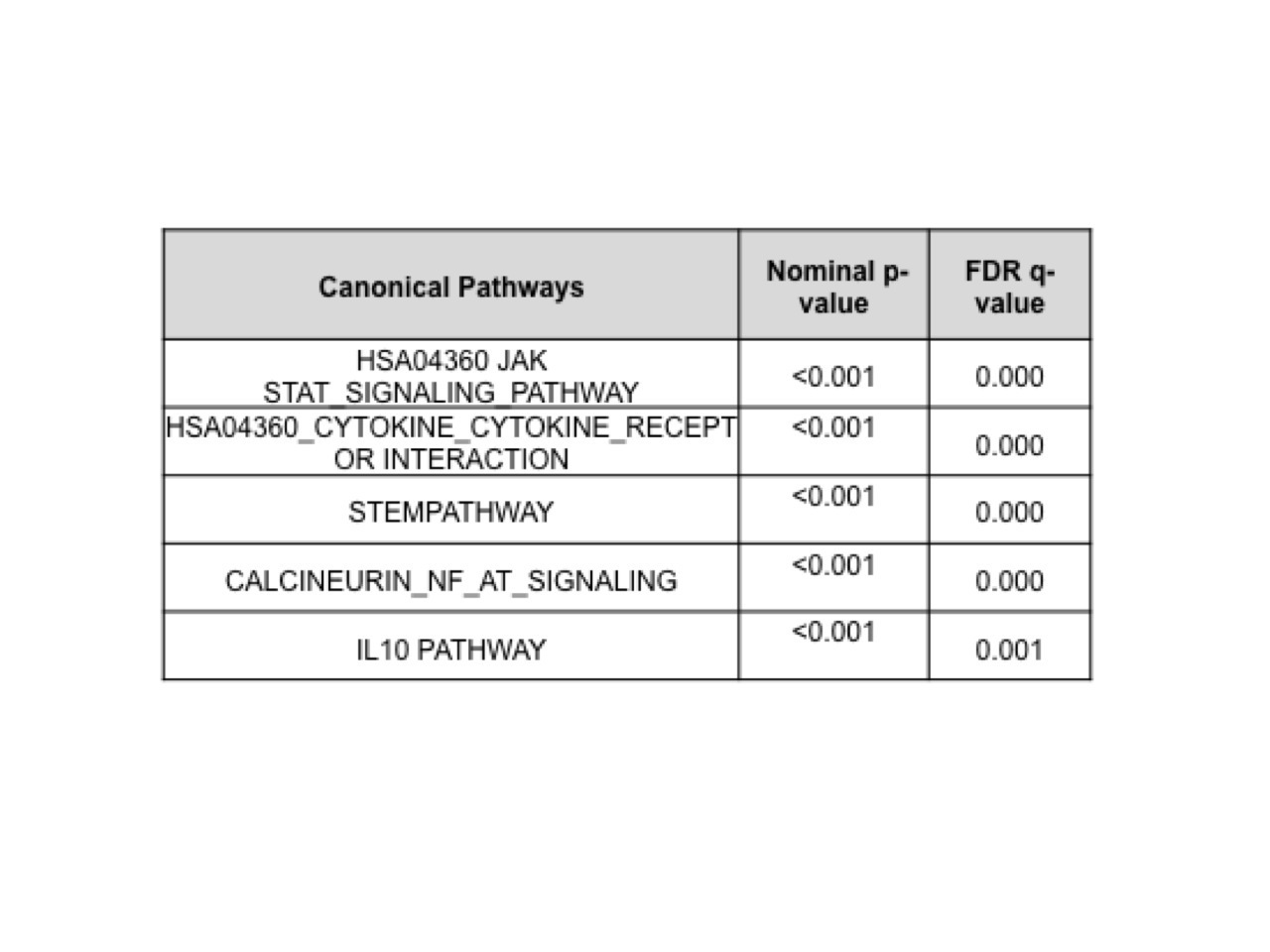

Supplement: Figure S7 — MAF1 alters immune signaling during type II infection. MEFs were infected with type II or type II:MAF1 Toxoplasma strains, or mock-infected. At 8 hpi, RNA was isolated from the cultures, and transcript abundance was determined using the Affymetrix Mouse 430 2.0 chip. Top 5 hits (ranked by normalized enrichment score) shown from GSEA_canonical pathway (c2.cp) analysis. (JPG) [file pbio.1001845.s007.jpg]

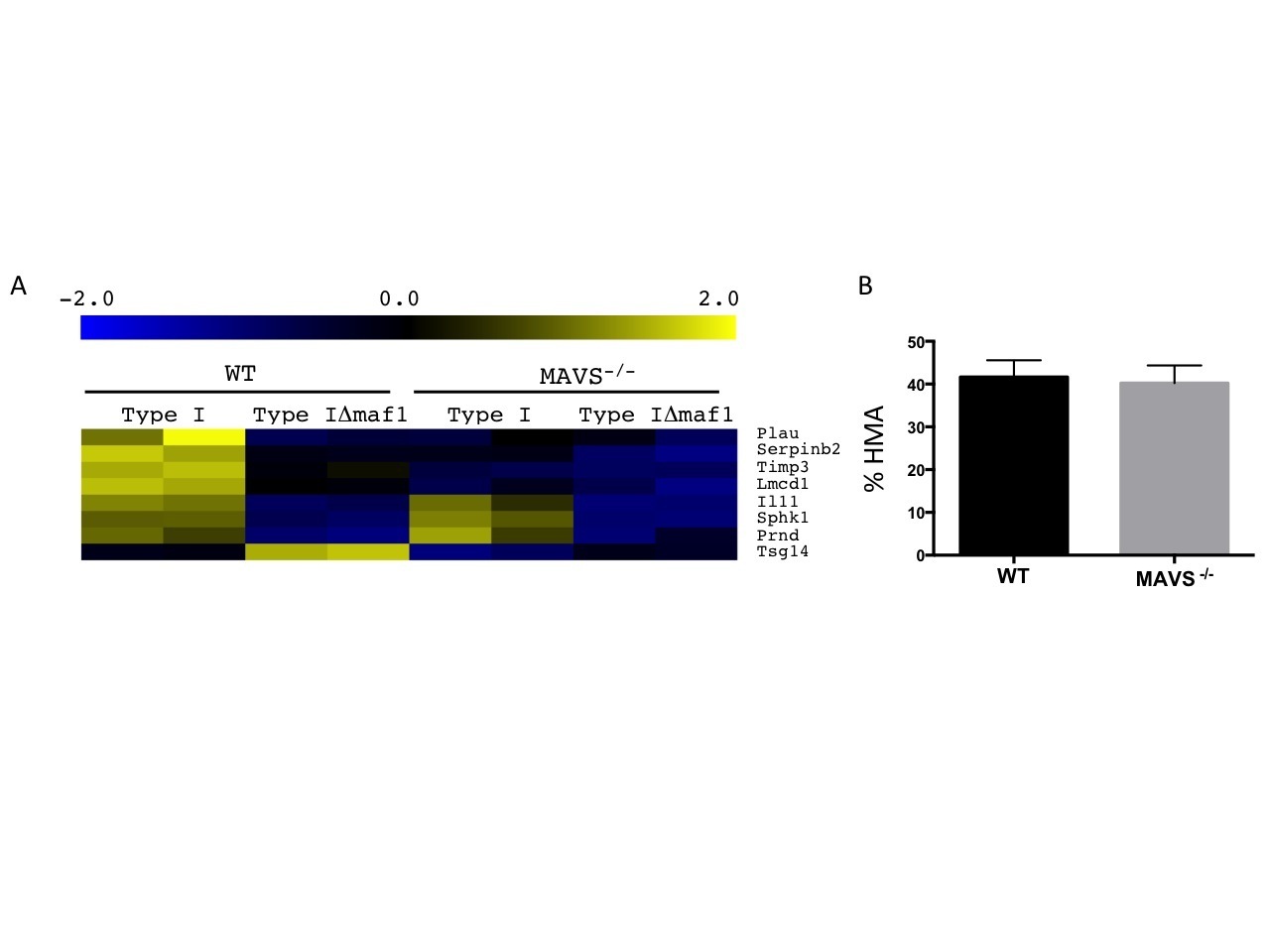

Supplement: Figure S8 — Role of MAVS in HMA signaling. (A) WT and MAVS−/− MEFs were infected with type I or type I:Δmaf1 Toxoplasma strains at an MOI of 5. At 8 hpi, RNA was isolated from the cultures, and transcript abundance was determined using the Affymetrix Mouse 430 2.0 chip. Genes whose expression was significantly different between type I infection and type I:Δmaf1 infection in WT MEFs are depicted. Colors indicate the deviation of each gene's signal above (yellow) and below (blue) its mean expression value across all eight samples. (B) Percentage of the PVM associated with mitochondria in WT and MAVS−/− MEFs infected with type I parasites (%HMA) as determined by ImageJ analysis of electron micrographs (n = 20 for each). Values shown are mean ± SEM. ****p<0.0001 using an unpaired t test. (JPG) [file pbio.1001845.s008.jpg]
